# Colorful Protein-Based Fluorescent Probes for Collagen Imaging

**DOI:** 10.1371/journal.pone.0114983

**Published:** 2014-12-09

**Authors:** Stijn J. A. Aper, Ariane C. C. van Spreeuwel, Mark C. van Turnhout, Ardjan J. van der Linden, Pascal A. Pieters, Nick L. L. van der Zon, Sander L. de la Rambelje, Carlijn V. C. Bouten, Maarten Merkx

**Affiliations:** 1 Laboratory of Chemical Biology and Institute of Complex Molecular Systems (ICMS), Department of Biomedical Engineering, Eindhoven University of Technology, MB Eindhoven, The Netherlands; 2 Soft Tissue Biomechanics and Engineering, Department of Biomedical Engineering, Eindhoven University of Technology, MB Eindhoven, The Netherlands; Institute of Dentistry, Barts & The London School of Medicine and Dentistry, Queen Mary University of London, United Kingdom

## Abstract

Real-time visualization of collagen is important in studies on tissue formation and remodeling in the research fields of developmental biology and tissue engineering. Our group has previously reported on a fluorescent probe for the specific imaging of collagen in live tissue *in situ*, consisting of the native collagen binding protein CNA35 labeled with fluorescent dye Oregon Green 488 (CNA35-OG488). The CNA35-OG488 probe has become widely used for collagen imaging. To allow for the use of CNA35-based probes in a broader range of applications, we here present a toolbox of six genetically-encoded collagen probes which are fusions of CNA35 to fluorescent proteins that span the visible spectrum: mTurquoise2, EGFP, mAmetrine, LSSmOrange, tdTomato and mCherry. While CNA35-OG488 requires a chemical conjugation step for labeling with the fluorescent dye, these protein-based probes can be easily produced in high yields by expression in *E. coli* and purified in one step using Ni^2+^-affinity chromatography. The probes all bind specifically to collagen, both *in vitro* and in porcine pericardial tissue. Some first applications of the probes are shown in multicolor imaging of engineered tissue and two-photon imaging of collagen in human skin. The fully-genetic encoding of the new probes makes them easily accessible to all scientists interested in collagen formation and remodeling.

## Introduction

Collagens form the most abundant protein family in vertebrates. As a major component of the extracellular matrix, collagen is essential for the load-bearing properties of tissue and provides a scaffold for cell attachment and growth [1]–[5]. Defects in collagen structure, production or degradation cause diseases such as chondrodysplasias, osteogenesis imperfecta, Ehlers Danlos syndrome, Alport syndrome, epidermolysis bullosa, osteoarthritis and osteoporosis [2]. Therefore, real-time imaging of collagen is important to increase understanding of the formation, orientation and remodeling of collagen in both diseased and healthy tissue, as studied in the fields of developmental biology, biomechanics and tissue engineering [1], [3]. Visualization of collagen has mostly been performed using techniques based on the intrinsic properties of collagen, like autofluorescence [6]–[8] and second harmonic generation [9]–[15]. The main advantage of these techniques is their non-invasiveness, i.e. the introduction of an exogenous probe is not required. However, their specificity is less well-defined since other tissue components also generate contrast. Furthermore, low signal intensities often limit contrast and resolution. Histological techniques such as picrosirius red staining [16] require cell fixation and therefore do not allow real-time monitoring of collagen.

Our group has previously reported the construction of a fluorescent probe that allows collagen imaging in live tissue based on the native collagen binding protein CNA35 [3]. CNA35 contains the first two soluble N1 and N2 domains of a collagen adhesion protein from *Staphylococcus aureus* bacteria. CNA35 recognizes the collagen triple helix according to the so-called “collagen hug model” [5]. Collagen first interacts with the concave trench of domain N2. Subsequently, domain N1 gets repositioned and stabilized, which induces the C-terminal latch of N2 to wrap around collagen and insert into the N1 domain. To allow CNA35 to be used as a collagen imaging probe the protein was labeled with the fluorescent dye Oregon Green 488 (OG488) via an amine-reactive succinimidyl ester resulting in 2–3 fluorescent labels per protein [3]. The probe was subsequently shown to bind to a variety of fibril-forming collagens including type I, III and IV, while no or little binding was observed to other common extracellular matrix proteins including laminin, elastin and fibronectin [3]. Collagen contains a high density of CNA35-binding sites with an average apparent *K*_d_ of 0.5 µM, which allows collagen to be stained readily, even without washing away excess probe. Finally, because binding of CNA35 to collagen is reversible, CNA35 is unlikely to interfere with extracellular matrix formation and thus provides an attractive probe for longitudinal studies in tissue engineering and tissue development.

Following our initial report in 2006 [3], CNA35-OG488 has become a popular collagen imaging probe, both in our own group and in those of our collaborators. In tissue engineering research CNA35-OG488 was used to evaluate the effect of mechanical and biochemical cues on extracellular matrix production, organization and remodeling in engineered, mostly cardiovascular tissue constructs [17]–[25]. Other applications of CNA35-OG488 include collagen fiber imaging in the adventitia of carotid arteries [26], [27] and visualization of the collagen architecture in atherosclerotic plaques [28]–[30], intervertebral disc tissue [31] and mammary gland [32]. In addition, the use of the probe as tool for clinical detection of atherosclerosis [29] and cardiovascular fibrosis [33] has been investigated. CNA35 has also been used in combination with other imaging modalities, for example via functionalization of paramagnetic micellar nanoparticles or liposomes for molecular MRI imaging of collagen in aortic aneurysms or atherosclerotic plaques [34]–[37]. Labeling gold nanoparticles with CNA35 or radioactive labeling allowed CT imaging or nuclear imaging of collagen [38], [39], and fusion of CNA35 to a luciferase led to immobilization of a luminescent reporter in the extracellular microenvironment [40]. Finally, our group reported the construction of a protease-activatable CNA35 probe as a dual-specific labeling agent [41], [42].

To further broaden the application of CNA35-based probes for collagen imaging we here report the construction of a toolbox of six genetically-encoded collagen probes consisting of fusions of CNA35 to a broad palette of fluorescent proteins that span the visible spectrum: mTurquoise2, EGFP, mAmetrine, LSSmOrange, tdTomato and mCherry. In contrast to CNA35-OG488, which requires a chemical conjugation step for labeling with the fluorescent dye, these protein-based probes can be easily produced in high yields (∼40 mg/L) by expression in *E. coli*, followed by a single purification step using Ni^2+^-affinity chromatography. The fully-genetic encoding of the new probes allows these probes to be easily shared, providing easy access of these tools to a much broader group of scientists. Successful collagen binding was demonstrated for all probes both in an *in vitro* collagen binding assay and in co-staining experiments on porcine pericardial tissue. First applications of these probes include their use in multicolor imaging of engineered tissue and two-photon imaging of collagen in human skin.

## Materials and Methods

### Cloning of expression plasmids

Plasmid pQE30-CNA35, encoding for domains N1 and N2 of the A-region of *Staphylococcus aureus* collagen adhesion protein (amino acids 30–344), was a kind gift from Magnus Höök (Texas A&M University, USA) [3], [5]. For cloning of the CNA35 gene into a pET28a vector we first performed PCR with primers CNA35_to_pET28a FW and RV (S1 Table) on pQE30-CNA35. Via the 5′-end of the forward primer, restriction sites *NheI* and *EcoRI* were introduced in front of the CNA35 gene and via the 5′-end of the reverse primer, restriction sites *AatII* and *XhoI* were included at the C-terminus of CNA35. Digestion of the PCR product at the distal end restriction sites *NheI* and *XhoI* - following the manufacturer’s instructions (New England Biolabs) - created a fragment that was compatible with a pET28a vector that had been treated with the same enzymes. Ligation was carried out at a 1∶5 vector-to-insert ratio using T4 DNA ligase (New England Biolabs) for 2 hours at 25°C, resulting in pET28a-CNA35. This strategy of cloning into pET28a led to the introduction of a 6xHis-tag in front of the CNA35 gene. The correct open reading frame of pET28a-CNA35 was confirmed by Sanger sequencing (StarSEQ GmbH, Mainz, Germany).

Plasmids pET28a-mCherry and pET28a-LSSmOrange were a kind gift from Laurens Lindenburg (Laboratory of Chemical Biology, Eindhoven University of Technology). The vectors for the other fluorescent proteins, pLifeAct-mTurquoise2 [43], pCSGreen-EGFP [44] and pmAmetrine-DEVD-tdTomato [45], were obtained from Addgene. The genes encoding for the fluorescent proteins were amplified from their vectors via PCR using the primers shown in S2 Table. For fusion of the fluorescent proteins to the N-terminus of CNA35, the restriction sites *NheI* and *EcoRI* were introduced before and after the fluorescent protein gene, respectively. Digestion of the PCR products - following the manufacturer’s instructions (New England Biolabs) - generated fragments that were compatible with pET28a-CNA35 that had been treated with the same enzymes. Ligations were performed at a 1∶5 vector-to-insert ratio using T4 DNA ligase (New England Biolabs) for 2 hours at 25°C, resulting in six vectors encoding for the N-terminal fusions of fluorescent protein and CNA35 (FP-CNA35). Fusion of the fluorescent proteins to the C-terminus of CNA35 was achieved via the same procedure as used for the N-terminal fusions, but with restriction enzymes *AatII* and *XhoI*, generating six plasmids encoding for the C-terminal fusions (CNA35-FP). The correct open reading frame of the generated vectors was confirmed by Sanger sequencing (StarSEQ GmbH, Mainz, Germany).

### Protein expression and purification

The plasmids encoding for the FP-CNA35 and CNA35-FP proteins were transformed into *E. coli* BL21(DE3) competent bacteria (Novagen). Single colonies were picked and used to inoculate 8 mL Luria-Bertani (LB) medium cultures (10 g/L peptone, 10 g/L NaCl, 5 g/L yeast extract) supplemented with 30 µg/mL kanamycin. The bacteria were grown overnight at 37°C and 250 rpm. Next morning the cells in the small cultures were transferred to 400 mL LB medium cultures containing 30 µg/mL kanamycin. The bacteria were again grown at 37°C and 250 rpm until the optical density at 600 nm reached ∼0.6. Then, expression was induced by adding 0.5 mM isopropyl β-D-1-thiogalactopyranoside (IPTG; Sigma-Aldrich). Protein expression was performed for ∼20 hours at 25°C and 250 rpm. Bacteria were harvested by centrifugation for 10 min at 10,000 g. The pellets were resuspended in 8 mL Bugbuster (Novagen) supplemented with 8 µL benzonase (Novagen) and incubated for ∼40 min at room temperature in order to extract the protein. Next, the Bugbuster suspension was centrifuged for 20 min at 16,000 g. The resulting soluble fraction containing the protein was further purified via Ni^2+^-affinity chromatography using the N-terminal 6xHis-tag. An Econo-Pac chromatography column (Bio-Rad) was filled with 4 mL Ni-NTA HisBind resin (Novagen). The resin was loaded with Ni^2+^ by the addition of 5 column volumes of charge buffer (50 mM NiSO_4_) and subsequently washed with 5 column volumes of wash buffer (20 mM Tris-HCl (pH 7.9), 0.5 M NaCl, 30 mM imidazole). The soluble fraction of the cell lysis was loaded onto the column, followed by column washing with 8 column volumes of wash buffer. The His-tagged protein was eluted using 4 column volumes of elution buffer (20 mM Tris-HCl (pH 7.9), 0.5 M NaCl, 500 mM imidazole). The elution fractions with protein were kept on ice and exposure of the proteins to light was minimized. As high concentrations of imidazole can induce protein aggregation, the buffer in which the protein was dissolved was changed to 50 mM Tris-HCl (pH 8.0), 100 mM NaCl by repeated concentration and dilution steps using the Amicon Ultra-4 Centrifugal Filter Units (Millipore) following the manufacturer’s instructions. Although not essential for its use as collagen probe, CNA35-tdTomato was further purified using size exclusion chromatography on a Sephacryl S-200 High Resolution column (GE Healthcare). Fractions that contained pure protein were pooled. The fusion proteins of CNA35 and mAmetrine, LSSmOrange or tdTomato were kept in the dark at 37°C for ∼15 hours to allow complete chromophore maturation (Table 1) [46], [47]. The fluorescent proteins mTurquoise2, EGFP and mCherry were already fully maturated after protein expression and purification. All twelve proteins were frozen in liquid nitrogen and stored at −80°C. Purity and correct molecular weight were analyzed on a 10% SDS-PAGE gel. Protein concentrations were determined using the measured absorption of the fluorescent proteins and their extinction coefficients [43], [46], [47] (Table 1). Correct molecular weight of the fusion proteins of CNA35 and tdTomato was confirmed using liquid chromatography quadrupole time-of-flight mass spectrometry.

**Table 1:** Properties of fluorescent proteins fused to CNA35 [43], [46], [47], [52].

Fluorescentprotein	Excitationmaximum (nm)	Emissionmaximum (nm)	Extinctioncoefficient (M-1cm-1)	Quantumyield	pKa	Maturation half-time at 37°C (h)
**mTurquoise2**	435	474	30,000	0.93	3.1	ND^a^
**EGFP**	488	507	56,000	0.60	6.0	ND^a^
**mAmetrine**	406	526	45,000	0.58	6.2	0.8
**LSSmOrange**	437	572	52,000	0.45	5.7	2.3
**tdTomato**	554	581	138,000	0.69	4.7	1
**mCherry**	587	610	72,000	0.22	<4.5	0.25

a not determined.

### Fluorescence spectroscopy

Fluorescence excitation and emission spectra of the proteins were recorded in 50 mM Tris (pH 8.0), 100 mM NaCl at 200 nM protein concentration on a Varian Cary Eclipse fluorescence spectrophotometer.

### Solid-phase collagen binding assay

Rat tail collagen type I (Sigma-Aldrich, cat. no. C7661) was dissolved to 1 mg/mL in 0.1 M acetic acid by vigorously shaking at room temperature. Before coating the selected wells of a 96-well plate, the collagen was diluted to 200 µg/mL in 50 mM Tris (pH 8.0), 100 mM NaCl. The pH neutralization leads to collagen matrix formation and efficient attachment to the bottom of the wells. In each well 50 µL of collagen was loaded, the plate was sealed with adhesive foil and incubation took place for ∼2.5 hours at 37°C. Next, the wells were emptied and washed four times with 200 µL of plate wash buffer (20 mM 4-(2-hydroxyethyl)-1-piperazineethanesulfonic acid (HEPES; pH 7.2), 150 mM NaCl). After each buffer addition the plate was shaken for 30 seconds at 350 rpm. The control wells and the uncoated areas of the bottom of the collagen-loaded wells were blocked by loading the wells with 180 µL of 2% (w/v) bovine serum albumin (BSA) in plate wash buffer. The plate was sealed again and incubation was performed for ∼2.5 hours at 37°C. Subsequently, all wells of the plate were washed four times with 200 µL plate wash buffer, including plate shaking after each buffer addition step. Dilutions of the collagen probes were prepared and added in 50 µL volume to both the collagen-loaded and the control wells. The plate was sealed and incubation was performed for 1–1.5 hours at room temperature. Finally, all wells were washed five times with 200 µL plate wash buffer, including plate shaking. The amount of collagen bound probe was determined by measuring fluorescence emission at the excitation maxima of the fluorescent proteins (excitation bandwidth 10 nm, emission bandwidth 5 nm) on a Tecan Safire fluorescence microplate reader. During fluorescence measurements all wells were filled with 100 µL plate wash buffer. The binding experiments of all probes with collagen were performed in triplicate, with the exception of CNA35-OG488 which was measured in duplicate. The control binding experiments with BSA were performed in duplicate. All data were normalized to the maximum emission intensity that was measured for a particular CNA35-FP fusion protein.

### Dual staining of porcine pericardial tissue

Porcine pericardial tissue was obtained from the slaughterhouse. Each tissue sample was incubated overnight with 1 µM of FP-CNA35 or CNA35-FP probe and 1 µM of CNA35-OG488 in 0.5 mL phosphate buffered saline (PBS; Sigma-Aldrich) in a 24-well plate on a shaking table, at room temperature. For the EGFP probes co-staining with CNA35-mTurquoise2 was performed, as spectra of OG488 and EGFP show too much overlap. Next day the tissue samples were washed once with PBS and subsequently transferred to a microscopy slide and covered with Mowiol 4–88 (Sigma-Aldrich) and cover glass.

Confocal microscopy (Leica TCS SP5 X) was used for imaging of the stained pericardial tissue samples. Wavelength ranges for excitation and emission were chosen around the maxima of the spectra of the fluorescent proteins (S3 Table). A few of the probes were excited using a white light laser. As this laser only allows excitation between 470 and 670 nm, most of the probes were excited using a two-photon laser (Chameleon Verdi 18W, 680<λ <1080 nm). The same settings were used for both N- and C-terminal fusion of each fluorescent domain to CNA35. All pictures were obtained using a HCX PL Apo CS 20x/0.7 objective (Leica). Signals were recorded using a hybrid APD/PMT detector (HyD, Leica) with gain at 100% (no signal amplification). Laser power was adjusted such that a minimal number of pixels in the images was saturated.

### Multicolor labeling of engineered tissue

Neonatal cardiomyocytes and cardiac fibroblasts were isolated from 1–3 day old C57/BL6 mouse hearts as described before [48]. Animal experiments were approved by the Animal Experiments Committee of the Leiden University Medical Center (DEC no. 12055) and conformed to the Guide for the Care and Use of Laboratory Animals as stated by the National Institutes of Health. Cells were cultured in culture medium consisting of high-glucose Dulbecco’s Modified Eagle Medium (Lonza), supplemented with 10% heat-inactivated fetal bovine serum (Greiner Bio-One), 1% L-glutamine and 1% penicillin/streptomycin before they were used for microtissue seeding. Microtissues were created by seeding the cardiac cells in a hydrogel suspension in a microfabricated tissue gauge (µTUG) as described before [49]. The hydrogel mixture was prepared by mixing 50% rat tail collagen type I (3.2 mg/mL; BD Biosciences), 39% culture medium, 3% 0.25 M NaOH, and 8% growth factor-reduced Matrigel (BD Biosciences). The cultured cells were trypsinized and resuspended in the hydrogel at a density of 10^6^ cells/mL. The cell-seeded gel polymerized in an incubator at 37°C, 5% CO_2_ for 10 minutes. Subsequently, culture medium was added to the microtissues and changed every 2–3 days.

One day before imaging 7 µM CellTracker Green (Invitrogen) and 1 µM of collagen probe CNA35-mTurquoise2 were added to the microtissue to visualize all cells and collagen, and incubation was performed overnight at 37°C. Prior to imaging, the microtissue was incubated for 30 minutes with 100 nM of active mitochondria probe tetramethylrhodamine methyl ester (TMRM; Invitrogen) to stain cardiomyocytes [50]. Next, the culture medium was refreshed and imaging was performed.

Excitation of CellTracker Green, CNA35-mTurquoise2 and TMRM was performed at 488 nm (white light laser), 870 nm (two-photon laser) and 543 nm (white light laser), respectively. Spectral emission windows were 498–543 nm, 454–494 nm and 551–597 nm for CellTracker Green, CNA35-mTurquoise2 and TMRM, respectively. Pictures were obtained using either HCX PL Apo CS 63x/1.2 water immersion objective or HCX PL Apo CS 10x/0.4 objective. Detector gain was set at 100% (no signal amplification) and laser power was adjusted such that a minimal number of pixels in the images was overexposed.

### Two-photon imaging of human skin tissue using CNA35-tdTomato

Human skin tissue from female patients undergoing abdominal plastic surgery was obtained from the Catharina Hospital Eindhoven as part of routine abdominal plastic surgery. Verbal consent was obtained from patients, which was documented by the doctors that obtained the materials. Tissues were handed over anonymously, without any patient-specific information except for gender. According to the Dutch medical scientific research with human subjects act (WMO), secondary use of patient material does not require review by a Medical Ethics Examination Committee. A previous protocol involving the same material was submitted to the Medical Ethical Examination Committee of the hospital, who confirmed that their permission was not required. Following excision the tissue was stored at −30°C until labeling was performed. In preparation of the labeling, the tissue was thawed and PBS was added. Tissue was incubated overnight with 1 µM of CNA35-OG488 and 1 µM of CNA35-tdTomato in 0.5 mL PBS (Sigma-Aldrich) in a 24-well plate on a shaking table, at room temperature. Next day the tissue samples were washed once with PBS and subsequently transferred to a microscope slide and covered with Mowiol 4–88 (Sigma-Aldrich) and cover glass.

Single-photon excitation using the white light laser was performed at 495 nm and 554 nm for CNA35-OG488 and CNA35-tdTomato, respectively. Two-photon excitation of CNA35-OG488 with the two-photon laser was performed at 990 nm, and at 1040 nm for CNA35-tdTomato, close to the excitation maximum of 1050 nm that had been reported for tdTomato for two-photon imaging [51]. Spectral emission windows were 502–541 nm for CNA35-OG488 and 563–619 nm for CNA35-tdTomato. All pictures were obtained using a HCX PL Apo CS 20x/0.7 objective (Leica). Detector gain was set at 100% (no signal amplification) and laser power was adjusted such that a minimal number of pixels in the images was overexposed.

## Results and Discussion

Six fluorescent proteins were chosen for fusion to CNA35 (Table 1). The green fluorescent protein EGFP was selected, because its spectral properties are similar to those of OG488 [46]. mCherry was included in the toolbox, as this protein is the most widely used and photostable protein in the class of red-shifted fluorescent proteins [46], [52]. The most blue-shifted fluorescent protein in the series, mTurquoise2, is a cyan fluorescent protein that has the highest quantum yield (0.93) ever measured for a monomeric fluorescent protein [43]. mAmetrine and LSSmOrange were chosen because of their large Stokes shift which allows excitation of multiple fluorescent probes at the same time [47]. The red fluorescent protein tdTomato has optimal properties for two-photon imaging due to its high quantum yield and cross-section value when excited at 1050 nm [51]. The fluorescent proteins in the toolbox cover emission wavelengths from 474 to 610 nm and are therefore compatible with many other fluorophores used for tissue staining. In addition, the spectral properties of the fluorescent proteins match well with lasers and detectors of currently used microscopes.

**Figure 1:**
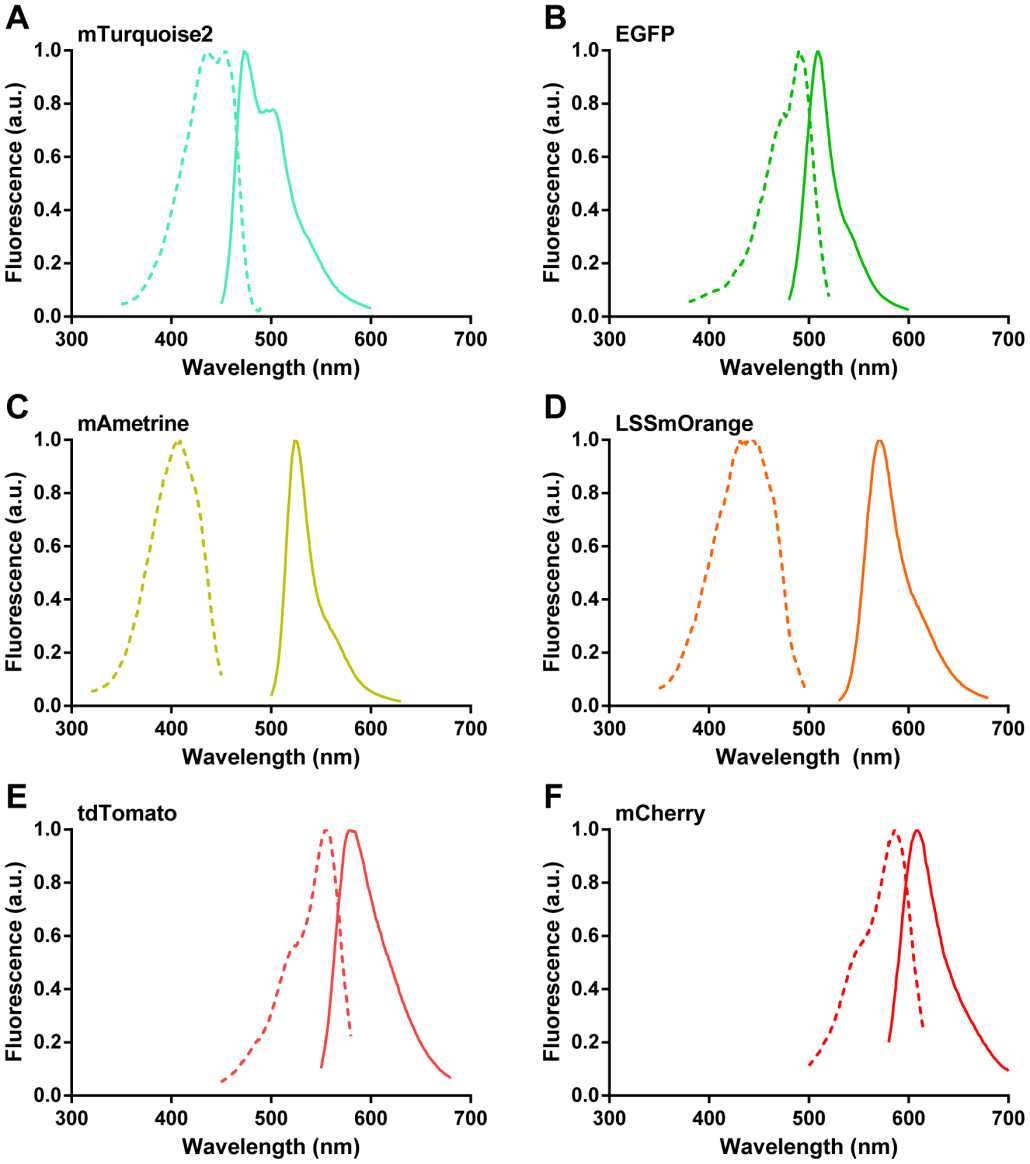
Spectra of fluorescent proteins fused to CNA35. Excitation (dotted line) and emission (solid line) spectra of CNA35 fusion proteins with mTurquoise2 (A), EGFP (B), mAmetrine (C), LSSmOrange (D), tdTomato (E) or mCherry (F). Spectra of the fluorescent proteins were similar for both N- and C-terminal fusion to CNA35. Measurements were performed in 50 mM Tris (pH 8.0), 100 mM NaCl.

We constructed fusions of the fluorescent proteins to either the N-terminus (FP-CNA35) or the C-terminus (CNA35-FP) of CNA35, which allowed us to investigate whether linkage to one of both ends of CNA35 would perturb its binding to collagen. We decided to introduce relatively long, flexible linkages between CNA35 and fluorescent protein (∼20 amino acids) to minimize perturbation of the fluorescent domains on the binding of CNA35 to collagen. The genes encoding for the fluorescent proteins were cloned at the N- or C-terminus of the CNA35 gene in a pET28a vector via a combination of PCR and traditional cloning (S1–S12 Figures). All proteins were successfully overexpressed in *E. coli* BL21(DE3). The soluble protein fraction was obtained via chemical lysis of the cells. Subsequently, the proteins were purified via Ni^2+^-affinity chromatography using their N-terminal His-tag. This one-step purification led to a high purity for the FP-CNA35 proteins, whereas the purity of the CNA35-FP probes was sufficient to allow application in collagen imaging (S13 Figure). The main impurity that remained for some of the CNA35-FP proteins was a cleavage product of CNA35 which is unlikely to interfere during collagen imaging. High expression yields of ∼40 mg/L were obtained for all probes, except for the large tdTomato fusion proteins which were obtained with yields of ∼10 mg/L. Since both CNA35 and the fluorescent domains are very stable proteins, the fusion proteins are also very stable and easy to handle. Fig. 1 shows that the excitation and emission spectra of the FP-CNA35 and CNA35-FP fusion proteins are the same as those reported for the individual domains [43], [46], [47], [51].

**Figure 2:**
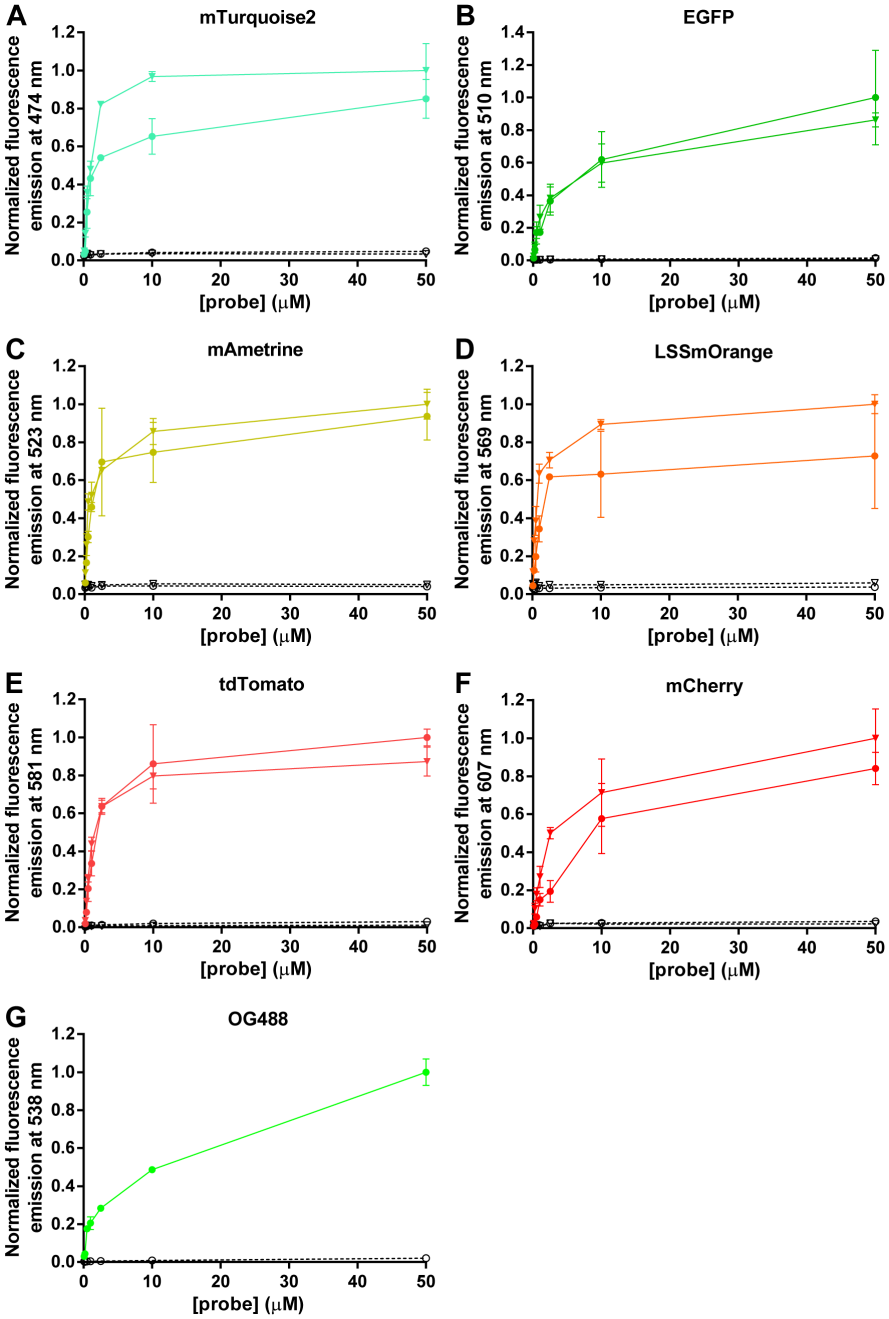
Solid-phase collagen binding of FP-CNA35 and CNA35-FP probes. Normalized fluorescence emission of FP-CNA35 and CNA35-FP probes bound to surface-immobilized rat tail collagen type I or BSA. mTurquoise2-CNA35 and CNA35-mTurquoise2 (A), EGFP-CNA35 and CNA35-EGFP (B), mAmetrine-CNA35 and CNA35-mAmetrine (C), LSSmOrange-CNA35 and CNA35-LSSmOrange (D), tdTomato-CNA35 and CNA35-tdTomato (E), mCherry-CNA35 and CNA35-mCherry (F), CNA35-OG488 (G) were loaded on collagen (closed circle for N-terminal fusion, closed triangle for C-terminal fusion) or BSA (open circle for N-terminal fusion, open triangle for C-terminal fusion) that were immobilized in wells of a 96-well plate. Emission of the fluorescent proteins was measured at the indicated wavelengths when excited at the excitation maxima (Table 1). Assays were performed in HEPES (pH 7.2), 150 mM NaCl. Binding of the FP-CNA35 and CNA35-FP probes to collagen was measured in triplicate. The binding experiments of all probes with BSA were performed in duplicate. Binding of CNA35-OG488 to collagen was also measured in duplicate. Error bars indicate ±1 SD.

**Figure 3:**
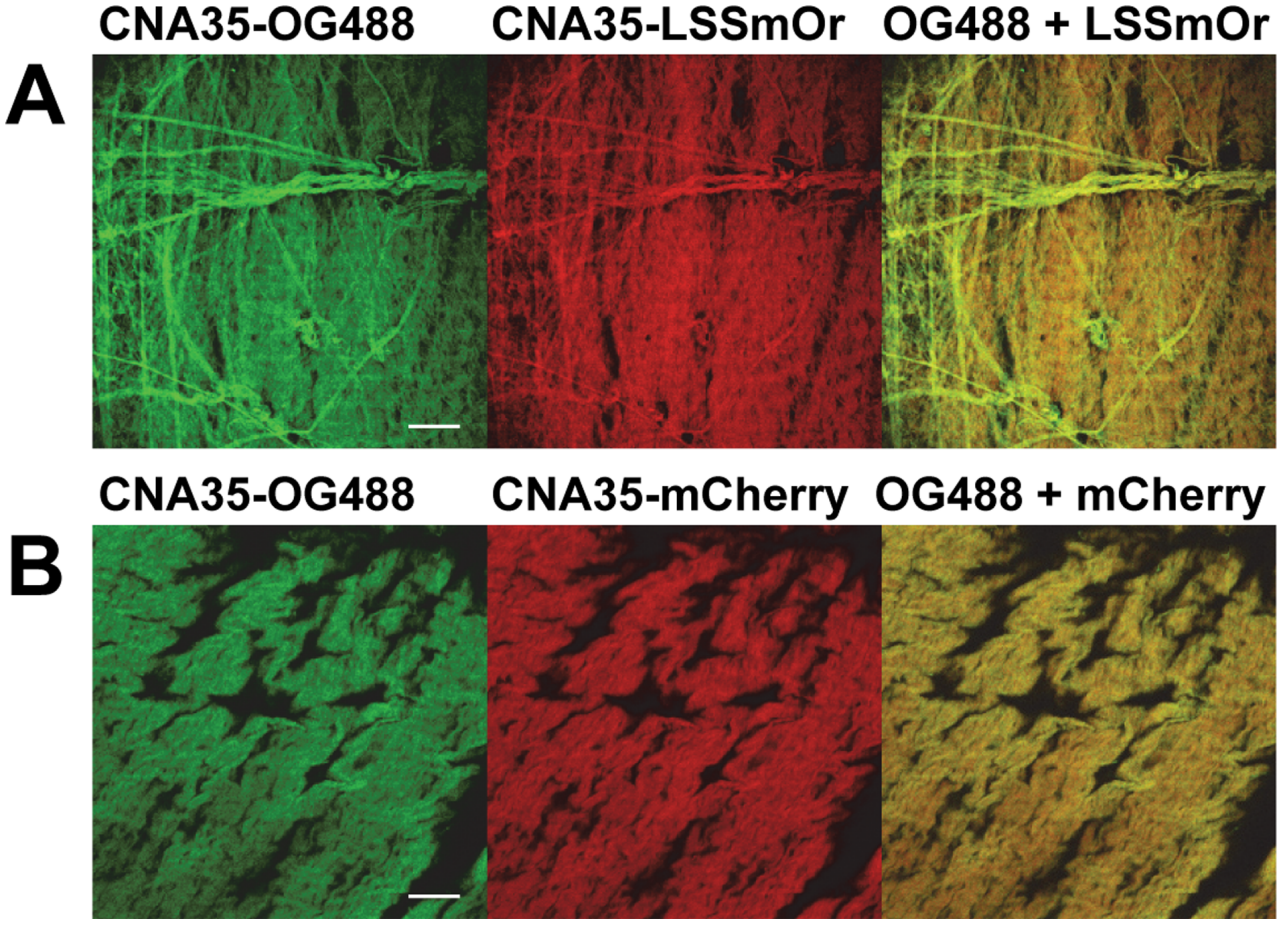
Dual staining of porcine pericardial tissue. Confocal fluorescence microscopy images showing dual staining of porcine pericardial tissue with CNA35-OG488 and CNA35-FP probes. (A) Images of collagen stained with 1 µM CNA35-OG488 and 1 µM CNA35-LSSmOrange, after overnight incubation of tissue with the probes in PBS at room temperature. Left: OG488 emission. Middle: LSSmOrange emission. Right: OG488+ LSSmOrange emission. Scale bar = 100 µm. (B) Images of collagen stained with 1 µM CNA35-OG488 and 1 µM CNA35-mCherry, after overnight incubation of tissue with the probes in PBS at room temperature. Left: OG488 emission. Middle: mCherry emission. Right: OG488+ mCherry emission. Scale bar = 100 µm. Excitation wavelengths and spectral emission windows used for imaging can be found in table S3.

To ensure that the probes still bind to collagen after fusion of CNA35 and fluorescent protein, solid-phase collagen binding assays were performed. A range of probe dilutions (0.1–50 µM) was loaded on rat tail collagen type I that was immobilized in wells of a 96-well plate. All probes showed clear binding to collagen, similar to binding observed for CNA35-OG488, with little to no binding in control wells that had been blocked with BSA (Fig. 2). Whereas collagen is known to contain both high and low affinity binding sites for CNA35, in these solid-phase binding assays all probes show a similar apparent overall *K*_d_ of ∼1 µM. Fusion of the fluorescent proteins to either the N- or C-terminus of CNA35 did not result in significantly different binding behavior of the probes, indicating that CNA35 is not very sensitive to modifications at its termini. Subsequently, we investigated binding of the probes to porcine pericardial tissue that is rich in collagen type I. Pericardial tissue was dual stained with CNA35-OG488 and each of the twelve probes. Because of the similar spectra of EGFP and OG488, the EGFP probes were co-stained with the mTurquoise2 probes. The collagen labeling with the FP-CNA35 and CNA35-FP proteins overlapped well with CNA35-OG488 staining, and imaging resolutions were comparable (Fig. 3, S14 and S15 Figures). Individual collagen fibers could be clearly distinguished with each of the new probes and showed good co-staining with CNA35-OG488, with the possible exception of high-density fibers that are occasionally observed. The latter seem to be stained more efficiently by the CNA35-OG488 probe (e.g. S14A Figure), possibly because its smaller size allows it to bind to sterically restricted binding sites within the fiber.

**Figure 4:**
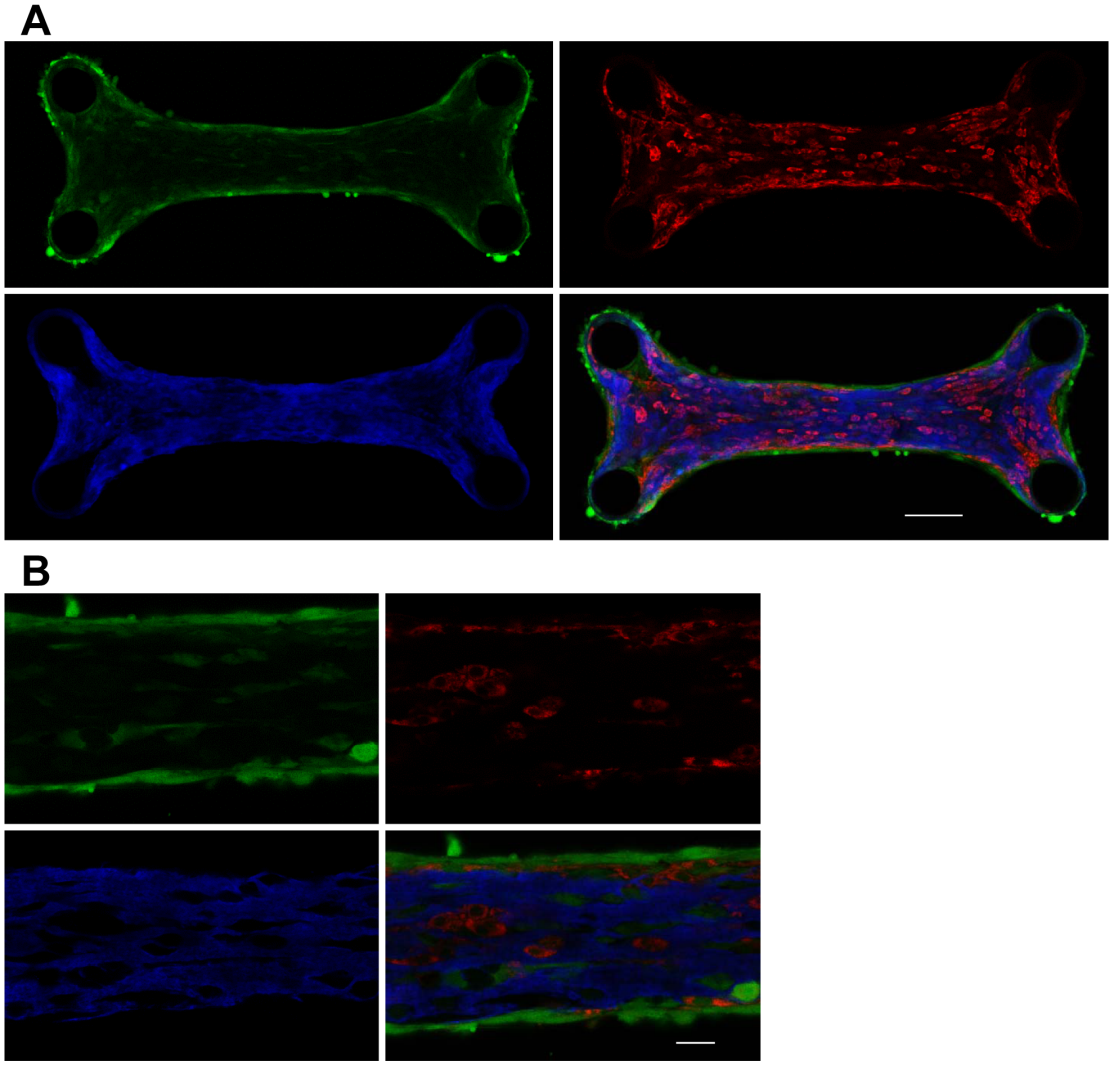
Multicolor labeling of engineered tissue. (A) Engineered collagen-rich tissue (thickness ≈ 50 µm) containing cardiomyocytes and cardiac fibroblasts stained with 7 µM CellTracker Green (left top image), 100 nM active mitochondria marker TMRM (right top) and 1 µM CNA35-mTurquoise2 (left bottom). The merged picture is shown at the right bottom. Incubation of the microtissue with CellTracker Green and CNA35-mTurquoise2 was done overnight at 37°C and TMRM was added 30 min before imaging. CellTracker Green labeled all cells (green), TMRM specifically stained cardiomyocytes (red) and collagen was stained with CNA35-mTurquoise2 (blue). Excitation of CellTracker Green, TMRM and CNA35-mTurquoise2 was performed at 488 nm, 543 nm and 870 nm, respectively, and spectral emission windows were 498–543 nm, 551–597 nm and 454–494 nm. Scale bar = 100 µm. (B) Higher magnification image of a region of the multicolor labeled tissue shown in (A). Scale bar = 25 µm.

To analyze the response of engineered tissue to mechanical and biochemical cues, a variety of tissue components including collagen needs to be stained [17]–[25]. The availability of six different colors for collagen imaging is attractive for such multicolor imaging applications, in particular since many commercially available cell markers are labeled with green or red fluorophores. Fig. 4 shows an example in which triple staining was applied to a collagen-rich microtissue containing cardiomyocytes and cardiac fibroblasts. All cells were stained with CellTracker Green, cardiomyocytes were stained with mitochondrial marker TMRM and collagen was labeled with CNA35-mTurquoise2. The different components of the engineered tissue were efficiently stained and easily distinguishable (Fig. 4a, b).

**Figure 5:**
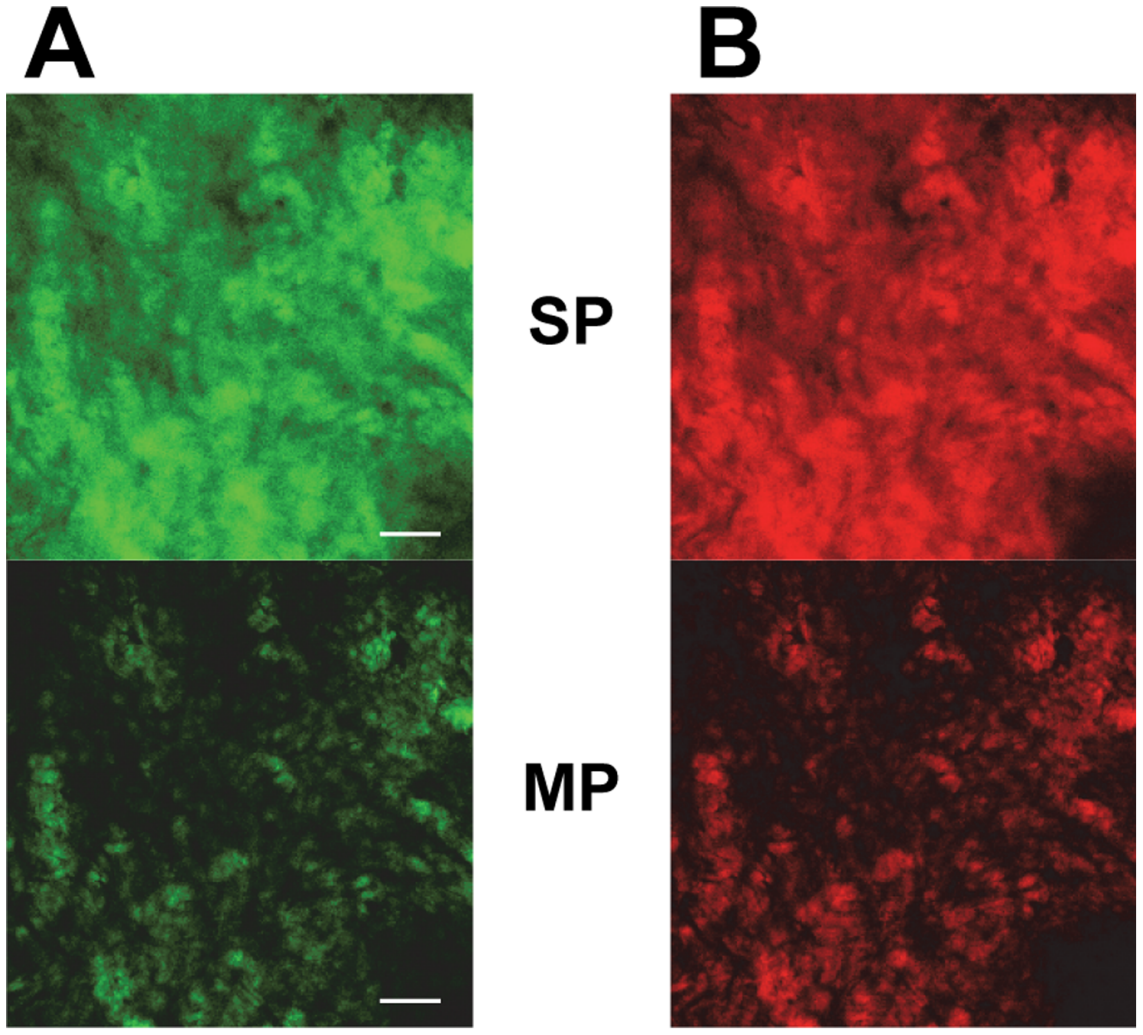
Two-photon imaging of human skin tissue using CNA35-tdTomato. (A) Single-photon (SP, top image; excitation at 495 nm) and two-photon imaging (MP, bottom image; excitation at 990 nm) of human skin tissue stained with 1 µM CNA35-OG488 at a depth of 50 µm, after overnight incubation of the tissue with the probe in PBS at room temperature. Spectral emission window was 502–541 nm. Scale bar = 100 µm. (B) Single-photon (SP, top image; excitation at 554 nm) and two-photon imaging (MP, bottom image; excitation at 1040 nm) of human skin tissue stained with 1 µM CNA35-tdTomato at a depth of 50 µm, after overnight incubation of the tissue with the probe in PBS at room temperature. Spectral emission window was 563–619 nm.

Research in tissue engineering and developmental biology often requires imaging of collagen at deeper tissue layers [7]. Two-photon imaging is especially useful for this application, as two-photon excitation allows deeper tissue penetration, diminishes autofluorescence and reduces out-of-focus photobleaching [51]. The red fluorescent protein tdTomato is one of the most optimal fluorescent proteins for two-photon imaging. As CNA35-tdTomato is a large protein (∼93 kDa) and therefore could have limited tissue penetration, the performance of CNA35-tdTomato in two-photon imaging was investigated on a biologically relevant tissue sample. We labeled human skin tissue with both CNA35-OG488 and CNA35-tdTomato and imaged at a depth of 50 µm. As can be seen in Fig. 5 two-photon imaging led to a much higher contrast compared to single-photon imaging, because of the clearly lower background fluorescence signal. Concluding, CNA35-tdTomato diffused well into the tissue and presents a valuable tool for two-photon imaging of collagen.

## Conclusions

A toolbox of six genetically-encoded fluorescent collagen probes - fusions of fluorescent proteins and collagen binding protein CNA35 - spanning the visible emission spectrum has been generated. The probes can be overexpressed in *E. coli* and purified in one step using Ni^2+^-affinity chromatography. The probes were all shown to bind specifically to collagen, both *in vitro* and in porcine pericardial tissue, performing at least as well as the previously reported chemically-labeled CNA35-OG488 probe. These newly developed probes are especially useful for real-time, multicolor labeling experiments of live tissue *in situ*. Their genetic encoding and straightforward *in vitro* production via recombinant expression in *E. coli* ensures their availability to all scientists interested in collagen imaging and in addition offers the possibility of direct probe expression in mammalian cells.

## Supporting Information

S1 Figure **Nucleotide sequence of bacterial expression vector pET28a-mTurquoise2-CNA35.** The DNA sequence is shown in lowercase, with the single letter amino acid code shown beneath each codon in uppercase. The His-tag is highlighted in green, the thrombin cleavage site in orange, mTurquoise2 in red and CNA35 in blue. Restriction sites for *NheI*, *EcoRI*, *AatII* and *XhoI* are shown italicized and underlined, and occur in the given order in the sequence from N- to C-terminus.
(PDF)

S2 Figure **Nucleotide sequence of bacterial expression vector pET28a-EGFP-CNA35.** The DNA sequence is shown in lowercase, with the single letter amino acid code shown beneath each codon in uppercase. The His-tag is highlighted in green, the thrombin cleavage site in orange, EGFP in red and CNA35 in blue. Restriction sites for *NheI*, *EcoRI*, *AatII* and *XhoI* are shown italicized and underlined, and occur in the given order in the sequence from N- to C-terminus.
(PDF)

S3 Figure **Nucleotide sequence of bacterial expression vector pET28a-mAmetrine-CNA35.** The DNA sequence is shown in lowercase, with the single letter amino acid code shown beneath each codon in uppercase. The His-tag is highlighted in green, the thrombin cleavage site in orange, mAmetrine in red and CNA35 in blue. Restriction sites for *NheI*, *EcoRI*, *AatII* and *XhoI* are shown italicized and underlined, and occur in the given order in the sequence from N- to C-terminus.
(PDF)

S4 Figure **Nucleotide sequence of bacterial expression vector pET28a-LSSmOrange-CNA35.** The DNA sequence is shown in lowercase, with the single letter amino acid code shown beneath each codon in uppercase. The His-tag is highlighted in green, the thrombin cleavage site in orange, LSSmOrange in red and CNA35 in blue. Restriction sites for *NheI*, *EcoRI*, *AatII* and *XhoI* are shown italicized and underlined, and occur in the given order in the sequence from N- to C-terminus.
(PDF)

S5 Figure **Nucleotide sequence of bacterial expression vector pET28a-tdTomato-CNA35.** The DNA sequence is shown in lowercase, with the single letter amino acid code shown beneath each codon in uppercase. The His-tag is highlighted in green, the thrombin cleavage site in orange, tdTomato in red and CNA35 in blue. Restriction sites for *NheI*, *EcoRI*, *AatII* and *XhoI* are shown italicized and underlined, and occur in the given order in the sequence from N- to C-terminus.
(PDF)

S6 Figure **Nucleotide sequence of bacterial expression vector pET28a-mCherry-CNA35.** The DNA sequence is shown in lowercase, with the single letter amino acid code shown beneath each codon in uppercase. The His-tag is highlighted in green, the thrombin cleavage site in orange, mCherry in red and CNA35 in blue. Restriction sites for *NheI*, *EcoRI*, *AatII* and *XhoI* are shown italicized and underlined, and occur in the given order in the sequence from N- to C-terminus.
(PDF)

S7 Figure **Nucleotide sequence of bacterial expression vector pET28a-CNA35-mTurquoise2.** The DNA sequence is shown in lowercase, with the single letter amino acid code shown beneath each codon in uppercase. The His-tag is highlighted in green, the thrombin cleavage site in orange, CNA35 in blue and mTurquoise2 in red. Restriction sites for *NheI*, *EcoRI*, *AatII* and *XhoI* are shown italicized and underlined, and occur in the given order in the sequence from N- to C-terminus.
(PDF)

S8 Figure **Nucleotide sequence of bacterial expression vector pET28a-CNA35-EGFP.** The DNA sequence is shown in lowercase, with the single letter amino acid code shown beneath each codon in uppercase. The His-tag is highlighted in green, the thrombin cleavage site in orange, CNA35 in blue and EGFP in red. Restriction sites for *NheI*, *EcoRI*, *AatII* and *XhoI* are shown italicized and underlined, and occur in the given order in the sequence from N- to C-terminus.
(PDF)

S9 Figure **Nucleotide sequence of bacterial expression vector pET28a-CNA35-mAmetrine.** The DNA sequence is shown in lowercase, with the single letter amino acid code shown beneath each codon in uppercase. The His-tag is highlighted in green, the thrombin cleavage site in orange, CNA35 in blue and mAmetrine in red. Restriction sites for *NheI*, *EcoRI*, *AatII* and *XhoI* are shown italicized and underlined, and occur in the given order in the sequence from N- to C-terminus.
(PDF)

S10 Figure **Nucleotide sequence of bacterial expression vector pET28a-CNA35-LSSmOrange.** The DNA sequence is shown in lowercase, with the single letter amino acid code shown beneath each codon in uppercase. The His-tag is highlighted in green, the thrombin cleavage site in orange, CNA35 in blue and LSSmOrange in red. Restriction sites for *NheI*, *EcoRI*, *AatII* and *XhoI* are shown italicized and underlined, and occur in the given order in the sequence from N- to C-terminus.
(PDF)

S11 Figure **Nucleotide sequence of bacterial expression vector pET28a-CNA35-tdTomato.** The DNA sequence is shown in lowercase, with the single letter amino acid code shown beneath each codon in uppercase. The His-tag is highlighted in green, the thrombin cleavage site in orange, CNA35 in blue and tdTomato in red. Restriction sites for *NheI*, *EcoRI*, *AatII* and *XhoI* are shown italicized and underlined, and occur in the given order in the sequence from N- to C-terminus.
(PDF)

S12 Figure **Nucleotide sequence of bacterial expression vector pET28a-CNA35-mCherry.** The DNA sequence is shown in lowercase, with the single letter amino acid code shown beneath each codon in uppercase. The His-tag is highlighted in green, the thrombin cleavage site in orange, CNA35 in blue and mCherry in red. Restriction sites for *NheI*, *EcoRI*, *AatII* and *XhoI* are shown italicized and underlined, and occur in the given order in the sequence from N- to C-terminus.
(PDF)

S13 Figure **SDS-PAGE analysis of purified FP-CNA35 and CNA35-FP probes.** 10% SDS-PAGE analysis of all probes. N = N-terminal fusion of fluorescent protein to CNA35. C = C-terminal fusion of fluorescent protein to CNA35. Molecular weights: mTurquoise2-CNA35 65.2 kDa; CNA35-mTurquoise2 65.0 kDa; EGFP-CNA35 65.0 kDa; CNA35-EGFP 65.1 kDa; mAmetrine-CNA35 64.3 kDa; CNA35-mAmetrine 64.4 kDa; LSSmOrange-CNA35 64.6 kDa; CNA35-LSSmOrange 64.7 kDa; tdTomato-CNA35 92.5 kDa; CNA35-tdTomato 93.1 kDa; mCherry-CNA35 64.6 kDa; CNA35-mCherry 64.7 kDa.
(TIF)

S14 Figure **Dual staining of porcine pericardial tissue with CNA35-OG488 and FP-CNA35 probes.** Confocal fluorescence microscopy images showing dual staining of porcine pericardial tissue with CNA35-OG488 and FP-CNA35 probes. Images of collagen stained with 1 µM CNA35-OG488 and 1 µM mTurquoise2-CNA35 (A), mAmetrine-CNA35 (C), LSSmOrange-CNA35 (D), tdTomato-CNA35 (E) and mCherry-CNA35 (F), after overnight incubation of tissue with the probes in PBS at room temperature. Left: OG488 emission. Middle: FP emission. Right: OG488+ FP emission. (B) EGFP-CNA35 was co-stained with mTurquoise2-CNA35, as spectra of OG488 and CNA35 show too much overlap. Excitation wavelengths and spectral emission windows used for imaging can be found in table S3. Scale bar = 100 µm.
(TIF)

S15 Figure **Dual staining of porcine pericardial tissue with CNA35-OG488 and CNA35-FP probes.** Confocal fluorescence microscopy images showing dual staining of porcine pericardial tissue with CNA35-OG488 and CNA35-FP probes. Images of collagen stained with 1 µM CNA35-OG488 and 1 µM CNA35-mTurquoise2 (A), CNA35-mAmetrine (C) and CNA35-tdTomato (D), after overnight incubation of tissue with the probes in PBS at room temperature. Left: OG488 emission. Middle: FP emission. Right: OG488+ FP emission. (B) CNA35-EGFP was co-stained with CNA35-mTurquoise2, as spectra of OG488 and EGFP show too much overlap. Excitation wavelengths and spectral emission windows used for imaging can be found in table S3. Scale bar = 100 µm.
(TIF)

S1 Table **Primers used for cloning of CNA35 gene into pET28a vector.** Restriction sites for *NheI* and *EcoRI* in primer CNA35_to_pET28a FW, and for *AatII* and *XhoI* in primer CNA35_to_pET28a RV are shown italicized and underlined.
(PDF)

S2 Table **Primers used for cloning fluorescent protein gene at N- or C-terminus of CNA35 gene.** In the primers used for constructing N-terminal fusions of fluorescent protein to CNA35, restriction sites for *NheI* and *EcoRI* are shown italicized and underlined in forward and reverse primer, respectively. In the primers used for constructing C-terminal fusions of fluorescent protein to CNA35, restriction sites for *AatII* and *XhoI* are shown italicized and underlined in forward and reverse primer, respectively.
(PDF)

S3 Table **Excitation and emission wavelengths used for imaging pericardial tissue stained with probe #1 and #2.**
(PDF)
