# Supplementary material for: Colorful Protein-Based Fluorescent Probes for Collagen Imaging
Source: PLoS One. 2014 Dec 9;9(12):e114983. doi: 10.1371/journal.pone.0114983 (PMC4260915; doi:10.1371/journal.pone.0114983)
Supplement: S1 Figure — Nucleotide sequence of bacterial expression vector pET28a-mTurquoise2-CNA35. The DNA sequence is shown in lowercase, with the single letter amino acid code shown beneath each codon in uppercase. The His-tag is highlighted in green, the thrombin cleavage site in orange, mTurquoise2 in red and CNA35 in blue. Restriction sites for NheI, EcoRI, AatII and XhoI are shown italicized and underlined, and occur in the given order in the sequence from N- to C-terminus. (PDF) [file pone.0114983.s001.pdf]

Figure S1. Nucleotide sequence of bacterial expression vector pET28a-mTurquoise2-CNA35

1 atgggcagcagccatcatcatcatcatcacagcagcggcctggtgccgcgcggcagccat

M G S S H H H H H H S S G L V P R G S H

61 atg*gctagc*ccggtcgccaccatggtgagcaagggcgaggagctgttcaccggggtggtg

M A S P V A T M V S K G E E L F T G V V

121 cccatcctggtcgagctggacggcgacgtaaacggccacaagttcagcgtgtccggcgag

P I L V E L D G D V N G H K F S V S G E

181 ggcgagggcgatgccacctacggcaagctgaccctgaagttcatctgcaccaccggcaag

G E G D A T Y G K L T L K F I C T T G K

241 ctgcccgtgccctggcccaccctcgtgaccaccctgtcctggggcgtgcagtgcttcgcc

L P V P W P T L V T T L S W G V Q C F A

301 cgctaccccgaccacatgaagcagcacgacttcttcaagtccgccatgcccgaaggctac

R Y P D H M K Q H D F F K S A M P E G Y

361 gtccaggagcgcaccatcttcttcaaggacgacggcaactacaagacccgcgccgaggtg

V Q E R T I F F K D D G N Y K T R A E V

421 aagttcgagggcgacaccctggtgaaccgcatcgagctgaagggcatcgacttcaaggag

K F E G D T L V N R I E L K G I D F K E

481 gacggcaacatcctggggcacaagctggagtacaactactttagcgacaacgtctatatc

D G N I L G H K L E Y N Y F S D N V Y I

541 accgccgacaagcagaagaacggcatcaaggccaacttcaagatccgccacaacatcgag

T A D K Q K N G I K A N F K I R H N I E

601 gacggcggcgtgcagctcgccgaccactaccagcagaacacccccatcggcgacggcccc

D G G V Q L A D H Y Q Q N T P I G D G P

661 gtgctgctgcccgacaaccactacctgagcacccagtccaagctgagcaaagaccccaac

V L L P D N H Y L S T Q S K L S K D P N

721 gagaagcgcgatcacatggtcctgctggagttcgtgaccgccgccgggatcactctcggc

E K R D H M V L L E F V T A A G I T L G

781 atggacgagctgtacaag*gaattc*cacggatccgcacgagatatttcatcaacgaatgtt

M D E L Y K E F H G S A R D I S S T N V

841 acagatttaactgtatcaccgtctaagatagaagatggtggtaaaacgacagtaaaaatg

T D L T V S P S K I E D G G K T T V K M

901 acgttcgacgataaaaatggaaaaatacaaaatggtgacatgattaaagtggcatggccg

T F D D K N G K I Q N G D M I K V A W P

961 acaagcggtacagtaaagatagagggttatagtaaaacagtaccattaactgttaaaggt

T S G T V K I E G Y S K T V P L T V K G

1021 gaacaggtgggtcaagcagttattacaccagacggtgcaacaattacattcaatgataaa

E Q V G Q A V I T P D G A T I T F N D K

1081 gtagaaaaattaagtgatgtttcgggatttgcagaatttgaagtacaaggaagaaattta

V E K L S D V S G F A E F E V Q G R N L

1141 acgcaaacaaatacttcagatgacaaagtagctacgataacatctgggaataaatcaacg

T Q T N T S D D K V A T I T S G N K S T

1201 aatgttacggttcataaaagtgaagcgggaacaagtagtgttttctattataaaacggga

N V T V H K S E A G T S S V F Y Y K T G

1261 gatatgctaccagaagatacgacacatgtacgatggtttttaaatattaacaatgaaaaa

D M L P E D T T H V R W F L N I N N E K

1321 agttatgtatcgaaagatattactataaaggatcagattcaaggtggacagcagttagat

S Y V S K D I T I K D Q I Q G G Q Q L D

1381 ttaagcacattaaacattaatgtgacaggtacacatagcaattattatagtggacaaagt

L S T L N I N V T G T H S N Y Y S G Q S

1441 gcaattactgattttgaaaaagcctttccaggttctaaaataactgttgataatacgaag

A I T D F E K A F P G S K I T V D N T K

1501 aacacaattgatgtaacaattccacaaggctatgggtcatataatagtttttcaattaac

N T I D V T I P Q G Y G S Y N S F S I N

1561 tacaaaaccaaaattacgaatgaacagcaaaaagagtttgttaataattcacaagcttgg

Y K T K I T N E Q Q K E F V N N S Q A W

1621 tatcaagagcatggtaaggaagaagtgaacgggaaatcatttaatcatactgtgcacaat

Y Q E H G K E E V N G K S F N H T V H N

1681 attaatgctaatgccggtattgaaggtactgtaaaaggtgaattaaaagttttaaaacag

I N A N A G I E G T V K G E L K V L K Q

1741 gataaagataccaaggcttca*gacgtc*ctgtaaggcattg*ctcgag*

D K D T K A S D V L -
