# Supplementary material for: Colorful Protein-Based Fluorescent Probes for Collagen Imaging
Source: PLoS One. 2014 Dec 9;9(12):e114983. doi: 10.1371/journal.pone.0114983 (PMC4260915; doi:10.1371/journal.pone.0114983)
Supplement: S2 Figure — Nucleotide sequence of bacterial expression vector pET28a-EGFP-CNA35. The DNA sequence is shown in lowercase, with the single letter amino acid code shown beneath each codon in uppercase. The His-tag is highlighted in green, the thrombin cleavage site in orange, EGFP in red and CNA35 in blue. Restriction sites for NheI, EcoRI, AatII and XhoI are shown italicized and underlined, and occur in the given order in the sequence from N- to C-terminus. (PDF) [file pone.0114983.s002.pdf]

Figure S2. Nucleotide sequence of bacterial expression vector pET28a-EGFP-CNA35

1 atgggcagcagccatcatcatcatcatcacagcagcggcctggtgccgcgcggcagccat

M G S S H H H H H H S S G L V P R G S H

61 atg*gctagc*atggtgagcaagggcgaggagctgttcaccggggtggtgcccatcctggtc

M A S M V S K G E E L F T G V V P I L V

121 gagctggacggcgacgtaaacggccacaagttcagcgtgtccggcgagggcgagggcgat

E L D G D V N G H K F S V S G E G E G D

181 gccacctacggcaagctgaccctgaagttcatctgcaccaccggcaagctgcccgtgccc

A T Y G K L T L K F I C T T G K L P V P

241 tggcccaccctcgtgaccaccctgacctacggcgtgcagtgcttcagccgctaccccgac

W P T L V T T L T Y G V Q C F S R Y P D

301 cacatgaagcagcacgacttcttcaagtccgccatgcccgaaggctacgtccaggagcgc

H M K Q H D F F K S A M P E G Y V Q E R

361 accatcttcttcaaggacgacggcaactacaagacccgcgccgaggtgaagttcgagggc

T I F F K D D G N Y K T R A E V K F E G

421 gacaccctggtgaaccgcatcgagctgaagggcatcgacttcaaggaggacggcaacatc

D T L V N R I E L K G I D F K E D G N I

481 ctggggcacaagctggagtacaactacaacagccacaacgtctatatcatggccgacaag

L G H K L E Y N Y N S H N V Y I M A D K

541 cagaagaacggcatcaaggtgaacttcaagatccgccacaacatcgaggacggcagcgtg

Q K N G I K V N F K I R H N I E D G S V

601 cagctcgccgaccactaccagcagaacacccccatcggcgacggccccgtgctgctgccc

Q L A D H Y Q Q N T P I G D G P V L L P

661 gacaaccactacctgagcacccagtccgccctgagcaaagaccccaacgagaagcgcgat

D N H Y L S T Q S A L S K D P N E K R D

721 cacatggtcctgctggagttcgtgaccgccgccgggatcactctcggcatggacgagctg

H M V L L E F V T A A G I T L G M D E L

781 tacaagtccgga*gaattc*cacggatccgcacgagatatttcatcaacgaatgttacagat

Y K S G E F H G S A R D I S S T N V T D

841 ttaactgtatcaccgtctaagatagaagatggtggtaaaacgacagtaaaaatgacgttc

L T V S P S K I E D G G K T T V K M T F

901 gacgataaaaatggaaaaatacaaaatggtgacatgattaaagtggcatggccgacaagc

D D K N G K I Q N G D M I K V A W P T S

961 ggtacagtaaagatagagggttatagtaaaacagtaccattaactgttaaaggtgaacag

G T V K I E G Y S K T V P L T V K G E Q

1021 gtgggtcaagcagttattacaccagacggtgcaacaattacattcaatgataaagtagaa

V G Q A V I T P D G A T I T F N D K V E

1081 aaattaagtgatgtttcgggatttgcagaatttgaagtacaaggaagaaatttaacgcaa

K L S D V S G F A E F E V Q G R N L T Q

1141 acaaatacttcagatgacaaagtagctacgataacatctgggaataaatcaacgaatgtt

T N T S D D K V A T I T S G N K S T N V

1201 acggttcataaaagtgaagcgggaacaagtagtgttttctattataaaacgggagatatg

T V H K S E A G T S S V F Y Y K T G D M

1261 ctaccagaagatacgacacatgtacgatggtttttaaatattaacaatgaaaaaagttat

L P E D T T H V R W F L N I N N E K S Y

1321 gtatcgaaagatattactataaaggatcagattcaaggtggacagcagttagatttaagc

V S K D I T I K D Q I Q G G Q Q L D L S

1381 acattaaacattaatgtgacaggtacacatagcaattattatagtggacaaagtgcaatt

T L N I N V T G T H S N Y Y S G Q S A I

1441 actgattttgaaaaagcctttccaggttctaaaataactgttgataatacgaagaacaca

T D F E K A F P G S K I T V D N T K N T

1501 attgatgtaacaattccacaaggctatgggtcatataatagtttttcaattaactacaaa

I D V T I P Q G Y G S Y N S F S I N Y K

1561 accaaaattacgaatgaacagcaaaaagagtttgttaataattcacaagcttggtatcaa

T K I T N E Q Q K E F V N N S Q A W Y Q

1621 gagcatggtaaggaagaagtgaacgggaaatcatttaatcatactgtgcacaatattaat

E H G K E E V N G K S F N H T V H N I N

1681 gctaatgccggtattgaaggtactgtaaaaggtgaattaaaagttttaaaacaggataaa

A N A G I E G T V K G E L K V L K Q D K

1741 gataccaaggcttca*gacgtc*ctgtaaggcattg*ctcgag*

D T K A S D V L -
