# Supplementary material for: Colorful Protein-Based Fluorescent Probes for Collagen Imaging
Source: PLoS One. 2014 Dec 9;9(12):e114983. doi: 10.1371/journal.pone.0114983 (PMC4260915; doi:10.1371/journal.pone.0114983)
Supplement: S3 Figure — Nucleotide sequence of bacterial expression vector pET28a-mAmetrine-CNA35. The DNA sequence is shown in lowercase, with the single letter amino acid code shown beneath each codon in uppercase. The His-tag is highlighted in green, the thrombin cleavage site in orange, mAmetrine in red and CNA35 in blue. Restriction sites for NheI, EcoRI, AatII and XhoI are shown italicized and underlined, and occur in the given order in the sequence from N- to C-terminus. (PDF) [file pone.0114983.s003.pdf]

Figure S3. Nucleotide sequence of bacterial expression vector pET28a-mAmetrine-CNA35

1 atgggcagcagccatcatcatcatcatcacagcagcggcctggtgccgcgcggcagccat
M G S S H H H H H H S S G L V P R G S H
61 atg*gctagc*ccggtcgccaccatggtgagcaagggcgaggagctgttcaccggggtggtg
M A S P V A T M V S K G E E L F T G V V
121 cccatcctggtcgagctggacggcgacgtaaacggccacaagttcagcgtgcgcggcgag
P I L V E L D G D V N G H K F S V R G E
181 ggcgagggcgatgccaccaacggcaagctgaccctgaagttcatctgcacctccggcaag
G E G D A T N G K L T L K F I C T S G K
241 ctgcccgtgccctggcccaccctcgtgaccaccctgtcttacggcgtgcagtgcttcgcc
L P V P W P T L V T T L S Y G V Q C F A
301 cgctaccccgaccacatgaagcagcacgacttcttcaagtccgccatgcccgaaggctac
R Y P D H M K Q H D F F K S A M P E G Y
361 gtccaggagcgcaccatctccttcaaggacgacggcagctacaggacccgcgccgaggtg
V Q E R T I S F K D D G S Y R T R A E V
421 aagttcgagggcgacaccctggtgaaccgcatcgagctgaagggcatcgacttcaaggag
K F E G D T L V N R I E L K G I D F K E
481 gacggcaacatcctggggcacaagctggagtacaacatgaacgtgtgggacgcgtatatc
D G N I L G H K L E Y N M N V W D A Y I
541 acggccgacaagcagaagaacggcatcaaagcgaacttcaagatcgagcacaacgtcgag
T A D K Q K N G I K A N F K I E H N V E
601 gacggcggcgtgcagctcgccgacgcgtaccagcagaacacccccatcggcgacggctcc
D G G V Q L A D A Y Q Q N T P I G D G S
661 gtgctgctgcctgacaaccactacctgagcttccagagcaagctgttcaaagaccccaac
V L L P D N H Y L S F Q S K L F K D P N
721 gagcagcgcgatcacatggtcctgctggagttcgttaccgccgccgggatcactctc*gaa*
E Q R D H M V L L E F V T A A G I T L E
781 *ttc*cacggatccgcacgagatatttcatcaacgaatgttacagatttaactgtatcaccg
F H G S A R D I S S T N V T D L T V S P
841 tctaagatagaagatggtggtaaaacgacagtaaaaatgacgttcgacgataaaaatgga
S K I E D G G K T T V K M T F D D K N G
901 aaaatacaaaatggtgacatgattaaagtggcatggccgacaagcggtacagtaaagata
K I Q N G D M I K V A W P T S G T V K I
961 gagggttatagtaaaacagtaccattaactgttaaaggtgaacaggtgggtcaagcagtt
E G Y S K T V P L T V K G E Q V G Q A V
1021 attacaccagacggtgcaacaattacattcaatgataaagtagaaaaattaagtgatgtt
I T P D G A T I T F N D K V E K L S D V
1081 tcgggatttgcagaatttgaagtacaaggaagaaatttaacgcaaacaaatacttcagat
S G F A E F E V Q G R N L T Q T N T S D
1141 gacaaagtagctacgataacatctgggaataaatcaacgaatgttacggttcataaaagt
D K V A T I T S G N K S T N V T V H K S
1201 gaagcgggaacaagtagtgttttctattataaaacgggagatatgctaccagaagatacg

E A G T S S V F Y Y K T G D M L P E D T

1261 acacatgtacgatggtttttaaatattaacaatgaaaaaagttatgtatcgaaagatatt

T H V R W F L N I N N E K S Y V S K D I

1321 actataaaggatcagattcaaggtggacagcagttagatttaagcacattaaacattaat

T I K D Q I Q G G Q Q L D L S T L N I N

1381 gtgacaggtacacatagcaattattatagtggacaaagtgcaattactgattttgaaaaa

V T G T H S N Y Y S G Q S A I T D F E K

1441 gcctttccaggttctaaaataactgttgataatacgaagaacacaattgatgtaacaatt

A F P G S K I T V D N T K N T I D V T I

1501 ccacaaggctatgggtcatataatagtttttcaattaactacaaaaccaaaattacgaat

P Q G Y G S Y N S F S I N Y K T K I T N

1561 gaacagcaaaaagagtttgttaataattcacaagcttggtatcaagagcatggtaaggaa

E Q Q K E F V N N S Q A W Y Q E H G K E

1621 gaagtgaacgggaaatcatttaatcatactgtgcacaatattaatgctaatgccggtatt

E V N G K S F N H T V H N I N A N A G I

1681 gaaggtactgtaaaaggtgaattaaaagttttaaaacaggataaagataccaaggcttca

E G T V K G E L K V L K Q D K D T K A S

1741 *gacgtc*ctgtaaggcattg*ctcgag*

D V L -
