# Supplementary material for: Colorful Protein-Based Fluorescent Probes for Collagen Imaging
Source: PLoS One. 2014 Dec 9;9(12):e114983. doi: 10.1371/journal.pone.0114983 (PMC4260915; doi:10.1371/journal.pone.0114983)
Supplement: S5 Figure — Nucleotide sequence of bacterial expression vector pET28a-tdTomato-CNA35. The DNA sequence is shown in lowercase, with the single letter amino acid code shown beneath each codon in uppercase. The His-tag is highlighted in green, the thrombin cleavage site in orange, tdTomato in red and CNA35 in blue. Restriction sites for NheI, EcoRI, AatII and XhoI are shown italicized and underlined, and occur in the given order in the sequence from N- to C-terminus. (PDF) [file pone.0114983.s005.pdf]

Figure S5. Nucleotide sequence of bacterial expression vector pET28a-tdTomato-CNA35

1 atgggcagcagccatcatcatcatcatcacagcagcggcctggtgccgcgcggcagccat
M G S S H H H H H H S S G L V P R G S H

61 atg*gctagc*gaggtcgatggtatggtgagcaagggcgaggaggtcatcaaagagttcatg
M A S E V D G M V S K G E E V I K E F M

121 cgcttcaaggtgcgcatggagggctccatgaacggccacgagttcgagatcgagggcgag
R F K V R M E G S M N G H E F E I E G E

181 ggcgagggccgcccctacgagggcacccagaccgccaagctgaaggtgaccaagggcggc
G E G R P Y E G T Q T A K L K V T K G G

241 cccctgcccttcgcctgggacatcctgtccccccagttcatgtacggctccaaggcgtac
P L P F A W D I L S P Q F M Y G S K A Y

301 gtgaagcaccccgccgacatccccgattacaagaagctgtccttccccgagggcttcaag
V K H P A D I P D Y K K L S F P E G F K

361 tgggagcgcgtgatgaacttcgaggacggcggtctggtgaccgtgacccaggactcctcc
W E R V M N F E D G G L V T V T Q D S S

421 ctgcaggacggcacgctgatctacaaggtgaagatgcgcggcaccaacttccccccgac
L Q D G T L I Y K V K M R G T N F P P D

481 ggccccgtaatgcagaagaagaccatgggctgggaggcctccaccgagcgcctgtacccc
G P V M Q K K T M G W E A S T E R L Y P

541 cgcgacggcgtgctgaagggcgagatccaccaggccctgaagctgaaggacggcggccac
R D G V L K G E I H Q A L K L K D G G H

601 tacctggtggagttcaagaccatctacatggccaagaagcccgtgcaactgcccggctac
Y L V E F K T I Y M A K K P V Q L P G Y

661 tactacgtggacaccaagctggacatcacctcccacaacgaggactacaccatcgtggaa
Y Y V D T K L D I T S H N E D Y T I V E

721 cagtacgagcgctccgagggccgccaccacctgttcctggggcatggcaccggcagcacc
Q Y E R S E G R H H L F L G H G T G S T

781 ggcagcggcagctccggcaccgcctcctccgaggacaacaacatggccgtcatcaaagag
G S G S S G T A S S E D N N M A V I K E

841 ttcatgcgcttcaaggtgcgcatggagggctccatgaacggccacgagttcgagatcgag
F M R F K V R M E G S M N G H E F E I E

901 ggcgagggcgagggccgcccctacgagggcacccagaccgccaagctgaaggtgaccaag
G E G E G R P Y E G T Q T A K L K V T K

961 ggcggccccctgcccttcgcctgggacatcctgtccccccagttcatgtacggctccaag
G G P L P F A W D I L S P Q F M Y G S K

1021 gcgtacgtgaagcaccccgccgacatccccgattacaagaagctgtccttccccgagggc
A Y V K H P A D I P D Y K K L S F P E G

1081 ttcaagtgggagcgcgtgatgaacttcgaggacggcggtctggtgaccgtgacccaggac
F K W E R V M N F E D G G L V T V T Q D

1141 tcctccctgcaggacggcacgctgatctacaaggtgaagatgcgcggcaccaacttcccc
S S L Q D G T L I Y K V K M R G T N F P

1201 cccgacggccccgtaatgcagaagaagaccatgggctgggaggcctccaccgagcgcctg

P D G P V M Q K K T M G W E A S T E R L

1261 taccccgcgacggcgtgctgaagggcgagatccaccaggccctgaagctgaaggacggc

Y P R D G V L K G E I H Q A L K L K D G

1321 ggccactacctggtggagttcaagaccatctacatggccaagaagcccgtgcaactgccc

G H Y L V E F K T I Y M A K K P V Q L P

1381 ggctactactacgtggacaccaagctggacatcacctcccacaacgaggactacaccatc

G Y Y Y V D T K L D I T S H N E D Y T I

1441 gtggaacagtacgagcgctccgagggccgccaccacctgttcctgtacggcatggacgag

V E Q Y E R S E G R H H L F L Y G M D E

1501 ctgtacaag*gaattc*cacggatccgcacgagatatttcatcaacgaatgttacagattta

L Y K E F H G S A R D I S S T N V T D L

1561 actgtatcaccgtctaagatagaagatggtggtaaaacgacagtaaaaatgacgttcgac

T V S P S K I E D G G K T T V K M T F D

1621 gataaaaatggaaaaatacaaaatggtgacatgattaaagtggcatggccgacaagcggt

D K N G K I Q N G D M I K V A W P T S G

1681 acagtaaagatagagggttatagtaaaacagtaccattaactgttaaaggtgaacaggtg

T V K I E G Y S K T V P L T V K G E Q V

1741 ggtcaagcagttattacaccagacggtgcaacaattacattcaatgataaagtagaaaaa

G Q A V I T P D G A T I T F N D K V E K

1801 ttaagtgatgtttcgggatttgcagaatttgaagtacaaggaagaaatttaacgcaaaca

L S D V S G F A E F E V Q G R N L T Q T

1861 aatacttcagatgacaaagtagctacgataacatctgggaataaatcaacgaatgttacg

N T S D D K V A T I T S G N K S T N V T

1921 gttcataaaagtgaagcgggaacaagtagtgtttctattataaaacgggagatatgcta

V H K S E A G T S S V F Y Y K T G D M L

1981 ccagaagatacgacacatgtacgatggttttaaatattaacaatgaaaaaagttatgta

P E D T T H V R W F L N I N N E K S Y V

2041 tcgaaagatattactataaaggatcagattcaaggtggacagcagttagatttaagcaca

S K D I T I K D Q I Q G G Q Q L D L S T

2101 ttaaacattaatgtgacaggtacacatagcaattattatagtggacaaagtgcaattact

L N I N V T G T H S N Y Y S G Q S A I T

2161 gattttgaaaaagcctttccaggttctaaaataactgttgataatacgaagaacacaatt

D F E K A F P G S K I T V D N T K N T I

2221 gatgtaacaattccacaaggctatgggtcatataatagtttttcaattaactacaaaacc

D V T I P Q G Y G S Y N S F S I N Y K T

2281 aaaattacgaatgaacagcaaaaagagtttgttaataattcacaagcttggtatcaagag

K I T N E Q Q K E F V N N S Q A W Y Q E

2341 catggtaaggaagaagtgaacgggaaatcatttaatcatactgtgcacaatattaatgct

H G K E E V N G K S F N H T V H N I N A

2401 aatgccggtattgaaggtactgtaaaaggtgaattaaaagttttaaaacaggataaagat

N A G I E G T V K G E L K V L K Q D K D

2461 accaaggcttca*gacgtc*ctgtaaggcattg*ctcgag*

T K A S D V L -
