# Supplementary material for: Colorful Protein-Based Fluorescent Probes for Collagen Imaging
Source: PLoS One. 2014 Dec 9;9(12):e114983. doi: 10.1371/journal.pone.0114983 (PMC4260915; doi:10.1371/journal.pone.0114983)
Supplement: S7 Figure — Nucleotide sequence of bacterial expression vector pET28a-CNA35-mTurquoise2. The DNA sequence is shown in lowercase, with the single letter amino acid code shown beneath each codon in uppercase. The His-tag is highlighted in green, the thrombin cleavage site in orange, CNA35 in blue and mTurquoise2 in red. Restriction sites for NheI, EcoRI, AatII and XhoI are shown italicized and underlined, and occur in the given order in the sequence from N- to C-terminus. (PDF) [file pone.0114983.s007.pdf]

Figure S7. Nucleotide sequence of bacterial expression vector pET28a-CNA35-mTurquoise2

1 atgggcagcagccatcatcatcatcatcacagcagcggcctggtgccgcgcggcagccat

M G S S H H H H H H S S G L V P R G S H

61 atg*gctagc*tcaggtgca*gaattc*cacggatccgcacgagatatttcatcaacgaatgtt

M A S S G A E F H G S A R D I S S T N V

121 acagatttaactgtatcaccgtctaagatagaagatggtggtaaaacgacagtaaaaatg

T D L T V S P S K I E D G G K T T V K M

181 acgttcgacgataaaaatggaaaaatacaaaatggtgacatgattaaagtggcatggccg

T F D D K N G K I Q N G D M I K V A W P

241 acaagcggtacagtaaagatagagggttatagtaaaacagtaccattaactgttaaaggt

T S G T V K I E G Y S K T V P L T V K G

301 gaacaggtgggtcaagcagttattacaccagacggtgcaacaattacattcaatgataaa

E Q V G Q A V I T P D G A T I T F N D K

361 gtagaaaaattaagtgatgtttcgggatttgcagaatttgaagtacaaggaagaaattta

V E K L S D V S G F A E F E V Q G R N L

421 acgcaaacaaatacttcagatgacaaagtagctacgataacatctgggaataaatcaacg

T Q T N T S D D K V A T I T S G N K S T

481 aatgttacggttcataaaagtgaagcgggaacaagtagtgttttctattataaaacggga

N V T V H K S E A G T S S V F Y Y K T G

541 gatatgctaccagaagatacgacacatgtacgatggtttttaaatattaacaatgaaaaa

D M L P E D T T H V R W F L N I N N E K

601 agttatgtatcgaaagatattactataaaggatcagattcaaggtggacagcagttagat

S Y V S K D I T I K D Q I Q G G Q Q L D

661 ttaagcacattaaacattaatgtgacaggtacacatagcaattattatagtggacaaagt

L S T L N I N V T G T H S N Y Y S G Q S

721 gcaattactgattttgaaaaagcctttccaggttctaaaataactgttgataatacgaag

A I T D F E K A F P G S K I T V D N T K

781 aacacaattgatgtaacaattccacaaggctatgggtcatataatagtttttcaattaac

N T I D V T I P Q G Y G S Y N S F S I N

841 tacaaaaccaaaattacgaatgaacagcaaaaagagtttgttaataattcacaagcttgg

Y K T K I T N E Q Q K E F V N N S Q A W

901 tatcaagagcatggtaaggaagaagtgaacgggaaatcatttaatcatactgtgcacaat

Y Q E H G K E E V N G K S F N H T V H N

961 attaatgctaatgccggtattgaaggtactgtaaaaggtgaattaaaagttttaaaacag

I N A N A G I E G T V K G E L K V L K Q

1021 gataaagataccaaggcttca*gacgtc*accatggtgagcaagggcgaggagctgttcacc

D K D T K A S D V T M V S K G E E L F T

1081 ggggtggtgcccatcctggtcgagctggacggcgacgtaaacggccacaagttcagcgtg

G V V P I L V E L D G D V N G H K F S V

1141 tccggcgagggcgagggcgatgccacctacggcaagctgaccctgaagttcatctgcacc

S G E G E G D A T Y G K L T L K F I C T

1201 accggcaagctgcccgtgccctggcccaccctcgtgaccaccctgtcctggggcgtgcag

T G K L P V P W P T L V T T L S W G V Q

1261 tgcttcgcccgctaccccgaccacatgaagcagcacgacttcttcaagtccgccatgccc

C F A R Y P D H M K Q H D F F K S A M P

1321 gaaggctacgtccaggagcgcaccatcttcttcaaggacgacggcaactacaagacccgc

E G Y V Q E R T I F F K D D G N Y K T R

1381 gccgaggtgaagttcgagggcgacaccctggtgaaccgcatcgagctgaagggcatcgac

A E V K F E G D T L V N R I E L K G I D

1441 ttcaaggaggacggcaacatcctggggcacaagctggagtacaactacttagcgacaac

F K E D G N I L G H K L E Y N Y F S D N

1501 gtctatatcaccgccgacaagcagaagaacggcatcaaggccaacttcaagatccgccac

V Y I T A D K Q K N G I K A N F K I R H

1561 aacatcgaggacggcggcgtgcagctcgccgaccactaccagcagaacacccccatcggc

N I E D G G V Q L A D H Y Q Q N T P I G

1621 gacggccccgtgctgctgcccgacaaccactacctgagcacccagtccaagctgagcaaa

D G P V L L P D N H Y L S T Q S K L S K

1681 gaccccaacgagaagcgcgatcacatggtcctgctggagttcgtgaccgccgccgggatc

D P N E K R D H M V L L E F V T A A G I

1741 actctcggcatggacgagctgtacaagtaagat*ctcgag*

T L G M D E L Y K -
