# Supplementary material for: Colorful Protein-Based Fluorescent Probes for Collagen Imaging
Source: PLoS One. 2014 Dec 9;9(12):e114983. doi: 10.1371/journal.pone.0114983 (PMC4260915; doi:10.1371/journal.pone.0114983)
Supplement: S9 Figure — Nucleotide sequence of bacterial expression vector pET28a-CNA35-mAmetrine. The DNA sequence is shown in lowercase, with the single letter amino acid code shown beneath each codon in uppercase. The His-tag is highlighted in green, the thrombin cleavage site in orange, CNA35 in blue and mAmetrine in red. Restriction sites for NheI, EcoRI, AatII and XhoI are shown italicized and underlined, and occur in the given order in the sequence from N- to C-terminus. (PDF) [file pone.0114983.s009.pdf]

Figure S9. Nucleotide sequence of bacterial expression vector pET28a-CNA35-mAmetrine

1 atgggcagcagccatcatcatcatcatcacagcagcggcctggtgccgcgcggcagccat

M G S S H H H H H H S S G L V P R G S H

61 atg*gctagc*tcaggtgca*gaattc*cacggatccgcacgagatatttcatcaacgaatgtt

M A S S G A E F H G S A R D I S S T N V

121 acagatttaactgtatcaccgtctaagatagaagatggtggtaaaacgacagtaaaaatg

T D L T V S P S K I E D G G K T T V K M

181 acgttcgacgataaaaatggaaaaatacaaaatggtgacatgattaaagtggcatggccg

T F D D K N G K I Q N G D M I K V A W P

241 acaagcggtacagtaaagatagagggttatagtaaaacagtaccattaactgttaaaggt

T S G T V K I E G Y S K T V P L T V K G

301 gaacaggtgggtcaagcagttattacaccagacggtgcaacaattacattcaatgataaa

E Q V G Q A V I T P D G A T I T F N D K

361 gtagaaaaattaagtgatgtttcgggatttgcagaatttgaagtacaaggaagaaattta

V E K L S D V S G F A E F E V Q G R N L

421 acgcaaacaaatacttcagatgacaaagtagctacgataacatctgggaataaatcaacg

T Q T N T S D D K V A T I T S G N K S T

481 aatgttacggttcataaaagtgaagcgggaacaagtagtgttttctattataaaacggga

N V T V H K S E A G T S S V F Y Y K T G

541 gatatgctaccagaagatacgacacatgtacgatggtttttaaatattaacaatgaaaaa

D M L P E D T T H V R W F L N I N N E K

601 agttatgtatcgaaagatattactataaaggatcagattcaaggtggacagcagttagat

S Y V S K D I T I K D Q I Q G G Q Q L D

661 ttaagcacattaaacattaatgtgacaggtacacatagcaattattatagtggacaaagt

L S T L N I N V T G T H S N Y Y S G Q S

721 gcaattactgattttgaaaaagcctttccaggttctaaaataactgttgataatacgaag

A I T D F E K A F P G S K I T V D N T K

781 aacacaattgatgtaacaattccacaaggctatgggtcatataatagttttcaattaac

N T I D V T I P Q G Y G S Y N S F S I N

841 tacaaaaccaaaattacgaatgaacagcaaaaagagtttgttaataattcacaagcttgg

Y K T K I T N E Q Q K E F V N N S Q A W

901 tatcaagagcatggtaaggaagaagtgaacgggaaatcatttaatcatactgtgcacaat

Y Q E H G K E E V N G K S F N H T V H N

961 attaatgctaatgccggtattgaaggtactgtaaaaggtgaattaaaagttttaaaacag

I N A N A G I E G T V K G E L K V L K Q

1021 gataaagataccaaggcttca*gacgtc*ccggtcgccaccatggtgagcaagggcgaggag

D K D T K A S D V P V A T M V S K G E E

1081 ctgttcaccggggtggtgcccatcctggtcgagctggacggcgacgtaaacggccacaag

L F T G V V P I L V E L D G D V N G H K

1141 ttcagcgtgcgcggcgagggcgagggcgatgccaccaacggcaagctgaccctgaagttc

F S V R G E G E G D A T N G K L T L K F

1201 atctgcacctccggcaagctgcccgtgccctggcccaccctcgtgaccaccctgtcttac

```
      I  C  T  S  G  K  L  P  V  P  W  P  T  L  V  T  T  L  S  Y
1261 ggcgtgcagtgcttcgcccgctaccccgaccacatgaagcagcacgacttcttcaagtcc
      G  V  Q  C  F  A  R  Y  P  D  H  M  K  Q  H  D  F  F  K  S
1321 gccatgcccgaaggctacgtccaggagcgcaccatctccttcaaggacgacggcagctac
      A  M  P  E  G  Y  V  Q  E  R  T  I  S  F  K  D  D  G  S  Y
1381 aggacccgcgccgaggtgaagttcgagggcgacaccctggtgaaccgcatcgagctgaag
      R  T  R  A  E  V  K  F  E  G  D  T  L  V  N  R  I  E  L  K
1441 ggcatcgacttcaaggaggacggcaacatcctggggcacaagctggagtacaacatgaac
      G  I  D  F  K  E  D  G  N  I  L  G  H  K  L  E  Y  N  M  N
1501 gtgtgggacgcgtatatcacggccgacaagcagaagaacggcatcaaagcgaacttcaag
      V  W  D  A  Y  I  T  A  D  K  Q  K  N  G  I  K  A  N  F  K
1561 atcgagcacaacgtcgaggacggcggcgtgcagctcgccgacgcgtaccagcagaacacc
      I  E  H  N  V  E  D  G  G  V  Q  L  A  D  A  Y  Q  Q  N  T
1621 cccatcggcgacggctccgtgctgctgcctgacaaccactacctgagcttccagagcaag
      P  I  G  D  G  S  V  L  L  P  D  N  H  Y  L  S  F  Q  S  K
1681 ctgttcaaagaccccaacgagcagcgcgatcacatggtcctgctggagttcgttaccgcc
      L  F  K  D  P  N  E  Q  R  D  H  M  V  L  L  E  F  V  T  A
1741 gccgggatcactctctaactcgag
      A  G  I  T  L  -
```
