# Supplementary material for: Colorful Protein-Based Fluorescent Probes for Collagen Imaging
Source: PLoS One. 2014 Dec 9;9(12):e114983. doi: 10.1371/journal.pone.0114983 (PMC4260915; doi:10.1371/journal.pone.0114983)
Supplement: S10 Figure — Nucleotide sequence of bacterial expression vector pET28a-CNA35-LSSmOrange. The DNA sequence is shown in lowercase, with the single letter amino acid code shown beneath each codon in uppercase. The His-tag is highlighted in green, the thrombin cleavage site in orange, CNA35 in blue and LSSmOrange in red. Restriction sites for NheI, EcoRI, AatII and XhoI are shown italicized and underlined, and occur in the given order in the sequence from N- to C-terminus. (PDF) [file pone.0114983.s010.pdf]

Figure S10. Nucleotide sequence of bacterial expression vector pET28a-CNA35-LSSmOrange

```
1     atgggcagcagccatcatcatcatcatcacagcagcggcctggtgccgcgcggcagccat
       M  G  S  S  H  H  H  H  H  H  S  S  G  L  V  P  R  G  S  H
61    atggctagctcaggtgcagaattccacggatccgcacgagatatttcatcaacgaatgtt
       M  A  S  S  G  A  E  F  H  G  S  A  R  D  I  S  S  T  N  V
121   acagatttaactgtatcaccgtctaagatagaagatggtggtaaaacgacagtaaaaatg
       T  D  L  T  V  S  P  S  K  I  E  D  G  G  K  T  T  V  K  M
181   acgttcgacgataaaaatggaaaaatacaaaatggtgacatgattaaagtggcatggccg
       T  F  D  D  K  N  G  K  I  Q  N  G  D  M  I  K  V  A  W  P
241   acaagcggtacagtaaagatagagggttatagtaaaacagtaccattaactgttaaaggt
       T  S  G  T  V  K  I  E  G  Y  S  K  T  V  P  L  T  V  K  G
301   gaacaggtgggtcaagcagttattacaccagacggtgcaacaattacattcaatgataaa
       E  Q  V  G  Q  A  V  I  T  P  D  G  A  T  I  T  F  N  D  K
361   gtagaaaaattaagtgatgtttcgggatttgcagaatttgaagtacaaggaagaaattta
       V  E  K  L  S  D  V  S  G  F  A  E  F  E  V  Q  G  R  N  L
421   acgcaaacaaatacttcagatgacaaagtagctacgataacatctgggaataaatcaacg
       T  Q  T  N  T  S  D  D  K  V  A  T  I  T  S  G  N  K  S  T
481   aatgttacggttcataaaagtgaagcgggaacaagtagtgttttctattataaaacggga
       N  V  T  V  H  K  S  E  A  G  T  S  S  V  F  Y  Y  K  T  G
541   gatatgctaccagaagatacgacacatgtacgatggttttaaatattaacaatgaaaaa
       D  M  L  P  E  D  T  T  H  V  R  W  F  L  N  I  N  N  E  K
601   agttatgtatcgaaagatattactataaaggatcagattcaaggtggacagcagttagat
       S  Y  V  S  K  D  I  T  I  K  D  Q  I  Q  G  G  Q  Q  L  D
661   ttaagcacattaaacattaatgtgacaggtacacatagcaattattatagtggacaaagt
       L  S  T  L  N  I  N  V  T  G  T  H  S  N  Y  Y  S  G  Q  S
721   gcaattactgattttgaaaaagcctttccaggttctaaaataactgttgataatacgaag
       A  I  T  D  F  E  K  A  F  P  G  S  K  I  T  V  D  N  T  K
781   aacacaattgatgtaacaattccacaaggctatgggtcatataatagttttcaattaac
       N  T  I  D  V  T  I  P  Q  G  Y  G  S  Y  N  S  F  S  I  N
841   tacaaaaccaaaattacgaatgaacagcaaaaagagtttgttaataattcacaagcttgg
       Y  K  T  K  I  T  N  E  Q  Q  K  E  F  V  N  N  S  Q  A  W
901   tatcaagagcatggtaaggaagaagtgaacgggaaatcatttaatcatactgtgcacaat
       Y  Q  E  H  G  K  E  E  V  N  G  K  S  F  N  H  T  V  H  N
961   attaatgctaatgccggtattgaaggtactgtaaaaggtgaattaaaagttttaaaacag
       I  N  A  N  A  G  I  E  G  T  V  K  G  E  L  K  V  L  K  Q
1021  gataaagataccaaggcttcagacgtcatggtgagcaaaggtgaggaaaacaatatggca
       D  K  D  T  K  A  S  D  V  M  V  S  K  G  E  E  N  N  M  A
1081  atcattaaagagtttatgcgtttcaaggtccgtatggaaggttctgtgaacggtcacgag
       I  I  K  E  F  M  R  F  K  V  R  M  E  G  S  V  N  G  H  E
1141  ttcgagattgaaggcgagggtgagggtcgtccgtatgagggcttccagactgttaaactg
       F  E  I  E  G  E  G  E  G  R  P  Y  E  G  F  Q  T  V  K  L
1201  aaggtgaccaaaggcggtcctctgccgttcgcctgggatatcctgtctccgcaattcaca
```

K V T K G G P L P F A W D I L S P Q F T

1261 tatgggtccaaggcgtatgttaaacaccctgcagatatccccgattatttaaaactgtca

Y G S K A Y V K H P A D I P D Y L K L S

1321 ttcccggaaggcttcaaatgggagcgtgtaatgaacttcgaggacggcggcgtcgtaacc

F P E G F K W E R V M N F E D G G V V T

1381 gttacccaggactcctctctgcaggacggtgagtttatttacaaagtgaagctgcgcggc

V T Q D S S L Q D G E F I Y K V K L R G

1441 actaactttccgtctgacggtccggttatgcagaaaaagactatgggcatggaagcttcc

T N F P S D G P V M Q K K T M G M E A S

1501 tctgaacgtatgtatccggaagacggcgcgctgaaaggtgaggacaaactgcgtctgaag

S E R M Y P E D G A L K G E D K L R L K

1561 ctgaaagatggcggtcattacacctctgaagttaaaaccacctacaaagccaaaaagccg

L K D G G H Y T S E V K T T Y K A K K P

1621 gtacaactgccgggtgcttacattgtagatattaaactggatatcacgagccataacgag

V Q L P G A Y I V D I K L D I T S H N E

1681 gattacacgattgttgaacagtacgaacgtgccgagggccgtcattctactggcggtatg

D Y T I V E Q Y E R A E G R H S T G G M

1741 gacgaactgtataaataa*ctcgag*

D E L Y K -
