# Supplementary material for: Colorful Protein-Based Fluorescent Probes for Collagen Imaging
Source: PLoS One. 2014 Dec 9;9(12):e114983. doi: 10.1371/journal.pone.0114983 (PMC4260915; doi:10.1371/journal.pone.0114983)
Supplement: S11 Figure — Nucleotide sequence of bacterial expression vector pET28a-CNA35-tdTomato. The DNA sequence is shown in lowercase, with the single letter amino acid code shown beneath each codon in uppercase. The His-tag is highlighted in green, the thrombin cleavage site in orange, CNA35 in blue and tdTomato in red. Restriction sites for NheI, EcoRI, AatII and XhoI are shown italicized and underlined, and occur in the given order in the sequence from N- to C-terminus. (PDF) [file pone.0114983.s011.pdf]

Figure S11. Nucleotide sequence of bacterial expression vector pET28a-CNA35-tdTomato

1 atgggcagcagccatcatcatcatcatcacagcagcggcctggtgccgcgcggcagccat
M G S S H H H H H H S S G L V P R G S H

61 atg*gctagc*tcaggtgca*gaattc*cacggatccgcacgagatatttcatcaacgaatgtt
M A S S G A E F H G S A R D I S S T N V

121 acagatttaactgtatcaccgtctaagatagaagatggtggtaaaacgacagtaaaaatg
T D L T V S P S K I E D G G K T T V K M

181 acgttcgacgataaaaatggaaaaatacaaaatggtgacatgattaaagtggcatggccg
T F D D K N G K I Q N G D M I K V A W P

241 acaagcggtacagtaaagatagagggttatagtaaaacagtaccattaactgttaaaggt
T S G T V K I E G Y S K T V P L T V K G

301 gaacaggtgggtcaagcagttattacaccagacggtgcaacaattacattcaatgataaa
E Q V G Q A V I T P D G A T I T F N D K

361 gtagaaaaattaagtgatgtttcgggatttgcagaatttgaagtacaaggaagaaattta
V E K L S D V S G F A E F E V Q G R N L

421 acgcaaacaaatacttcagatgacaaagtagctacgataacatctgggaataaatcaacg
T Q T N T S D D K V A T I T S G N K S T

481 aatgttacggttcataaaagtgaagcgggaacaagtagtgttttctattataaaacggga
N V T V H K S E A G T S S V F Y Y K T G

541 gatatgctaccagaagatacgacacatgtacgatggttttaaatattaacaatgaaaaa
D M L P E D T T H V R W F L N I N N E K

601 agttatgtatcgaaagatattactataaaggatcagattcaaggtggacagcagttagat
S Y V S K D I T I K D Q I Q G G Q Q L D

661 ttaagcacattaaacattaatgtgacaggtacacatagcaattattatagtggacaaagt
L S T L N I N V T G T H S N Y Y S G Q S

721 gcaattactgattttgaaaaagcctttccaggttctaaaataactgttgataatacgaag
A I T D F E K A F P G S K I T V D N T K

781 aacacaattgatgtaacaattccacaaggctatgggtcatataatagttttcaattaac
N T I D V T I P Q G Y G S Y N S F S I N

841 tacaaaaccaaaattacgaatgaacagcaaaaagagtttgttaataattcacaagcttgg
Y K T K I T N E Q Q K E F V N N S Q A W

901 tatcaagagcatggtaaggaagaagtgaacgggaaatcatttaatcatactgtgcacaat
Y Q E H G K E E V N G K S F N H T V H N

961 attaatgctaatgccggtattgaaggtactgtaaaaggtgaattaaaagttttaaaacag
I N A N A G I E G T V K G E L K V L K Q

1021 gataaagataccaaggcttca*gacgtc*ggcagtggcagcggagatgaggtcgatggtatg
D K D T K A S D V G S G S G D E V D G M

1081 gtgagcaagggcgaggaggtcatcaaagagttcatgcgcttcaaggtgcgcatggagggc
V S K G E E V I K E F M R F K V R M E G

1141 tccatgaacggccacgagttcgagatcgagggcgagggcgagggccgcccctacgagggc
S M N G H E F E I E G E G E G R P Y E G

1201 acccagaccgccaagctgaaggtgaccaagggcggccccctgcccttcgcctgggacatc

T Q T A K L K V T K G G P L P F A W D I

1261 ctgtcccccagttcatgtacggctccaaggcgtacgtgaagcaccccgccgacatcccc

L S P Q F M Y G S K A Y V K H P A D I P

1321 gattacaagaagctgtccttccccgagggcttcaagtgggagcgcgtgatgaacttcgag

D Y K K L S F P E G F K W E R V M N F E

1381 gacggcggtctggtgaccgtgacccaggactcctccctgcaggacggcacgctgatctac

D G G L V T V T Q D S S L Q D G T L I Y

1441 aaggtgaagatgcgcggcaccaacttcccccccgacggccccgtaatgcagaagaagacc

K V K M R G T N F P P D G P V M Q K K T

1501 atgggctgggaggcctccaccgagcgcctgtacccccgcgacggcgtgctgaagggcgag

M G W E A S T E R L Y P R D G V L K G E

1561 atccaccaggccctgaagctgaaggacggcggccactacctggtggagttcaagaccatc

I H Q A L K L K D G G H Y L V E F K T I

1621 tacatggccaagaagcccgtgcaactgcccggctactactacgtggacaccaagctggac

Y M A K K P V Q L P G Y Y Y V D T K L D

1681 atcacctcccacaacgaggactacaccatcgtggaacagtacgagcgctccgagggccgc

I T S H N E D Y T I V E Q Y E R S E G R

1741 caccacctgttcctggggcatggcaccggcagcaccggcagcggcagctccggcaccgcc

H H L F L G H G T G S T G S G S S G T A

1801 tcctccgaggacaacaacatggccgtcatcaaagagttcatgcgcttcaaggtgcgcatg

S S E D N N M A V I K E F M R F K V R M

1861 gagggctccatgaacggccacgagttcgagatcgagggcgagggcgagggccgcccctac

E G S M N G H E F E I E G E G E G R P Y

1921 gagggcacccagaccgccaagctgaaggtgaccaagggcggccccctgcccttcgcctgg

E G T Q T A K L K V T K G G P L P F A W

1981 gacatcctgtcccccagttcatgtacggctccaaggcgtacgtgaagcaccccgccgac

D I L S P Q F M Y G S K A Y V K H P A D

2041 atccccgattacaagaagctgtccttccccgagggcttcaagtgggagcgcgtgatgaac

I P D Y K K L S F P E G F K W E R V M N

2101 ttcgaggacggcggtctggtgaccgtgacccaggactcctccctgcaggacggcacgctg

F E D G G L V T V T Q D S S L Q D G T L

2161 atctacaaggtgaagatgcgcggcaccaacttcccccccgacggccccgtaatgcagaag

I Y K V K M R G T N F P P D G P V M Q K

2221 aagaccatgggctgggaggcctccaccgagcgcctgtacccccgcgacggcgtgctgaag

K T M G W E A S T E R L Y P R D G V L K

2281 ggcgagatccaccaggccctgaagctgaaggacggcggccactacctggtggagttcaag

G E I H Q A L K L K D G G H Y L V E F K

2341 accatctacatggccaagaagcccgtgcaactgcccggctactactacgtggacaccaag

T I Y M A K K P V Q L P G Y Y Y V D T K

2401 ctggacatcacctcccacaacgaggactacaccatcgtggaacagtacgagcgctccgag

L D I T S H N E D Y T I V E Q Y E R S E

2461 ggccgccaccacctgttcctgtacggcatggacgagctgtacaagtaacgat*ctcgag*

G R H H L F L Y G M D E L Y K -
