# Supplementary figures and images for: Colorful Protein-Based Fluorescent Probes for Collagen Imaging
Source: PLoS One. 2014 Dec 9;9(12):e114983. doi: 10.1371/journal.pone.0114983 (PMC4260915; doi:10.1371/journal.pone.0114983)

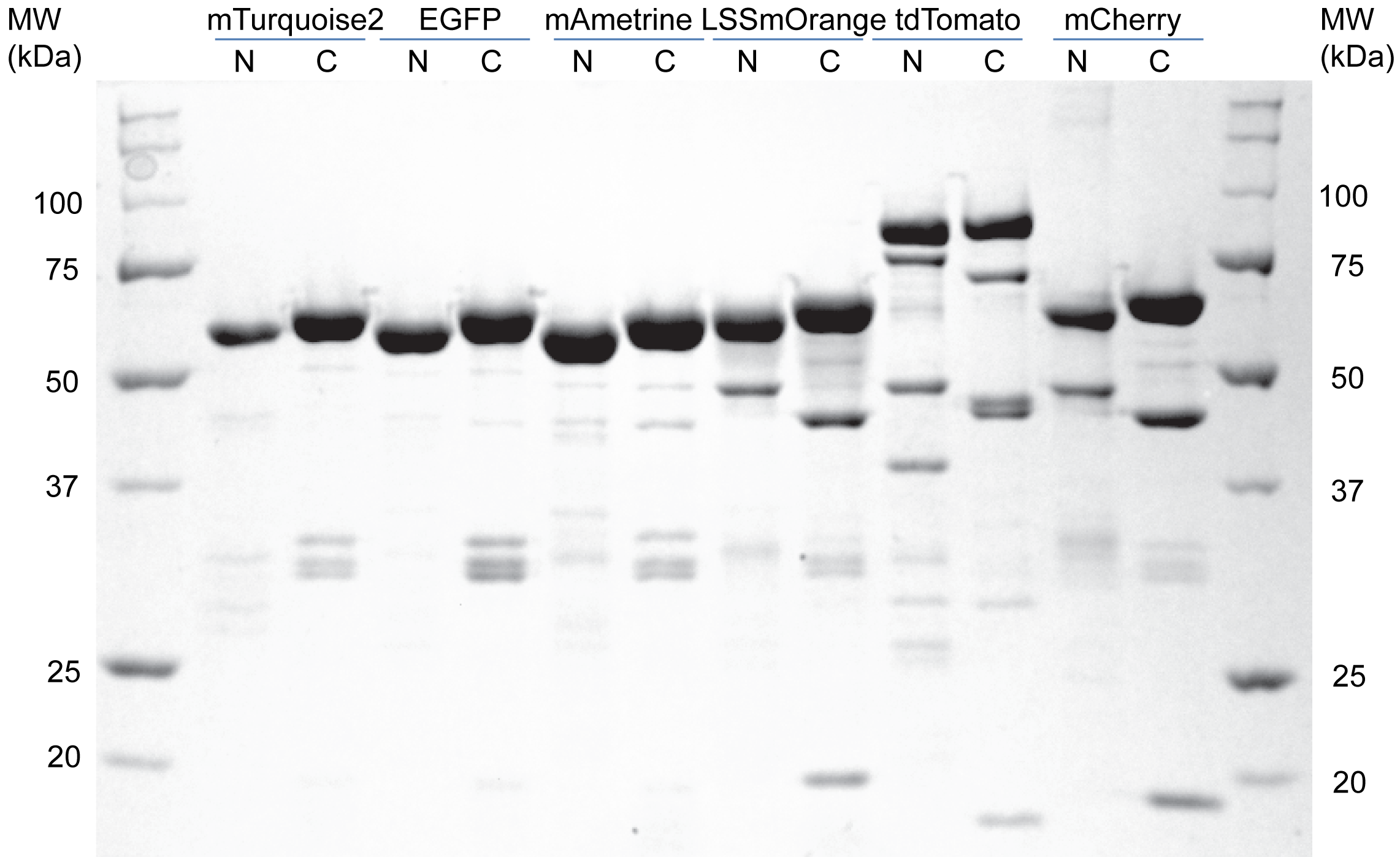

Supplement: S13 Figure — SDS-PAGE analysis of purified FP-CNA35 and CNA35-FP probes. 10% SDS-PAGE analysis of all probes. N = N-terminal fusion of fluorescent protein to CNA35. C = C-terminal fusion of fluorescent protein to CNA35. Molecular weights: mTurquoise2-CNA35 65.2 kDa; CNA35-mTurquoise2 65.0 kDa; EGFP-CNA35 65.0 kDa; CNA35-EGFP 65.1 kDa; mAmetrine-CNA35 64.3 kDa; CNA35-mAmetrine 64.4 kDa; LSSmOrange-CNA35 64.6 kDa; CNA35-LSSmOrange 64.7 kDa; tdTomato-CNA35 92.5 kDa; CNA35-tdTomato 93.1 kDa; mCherry-CNA35 64.6 kDa; CNA35-mCherry 64.7 kDa. (TIF) [file pone.0114983.s013.tif]

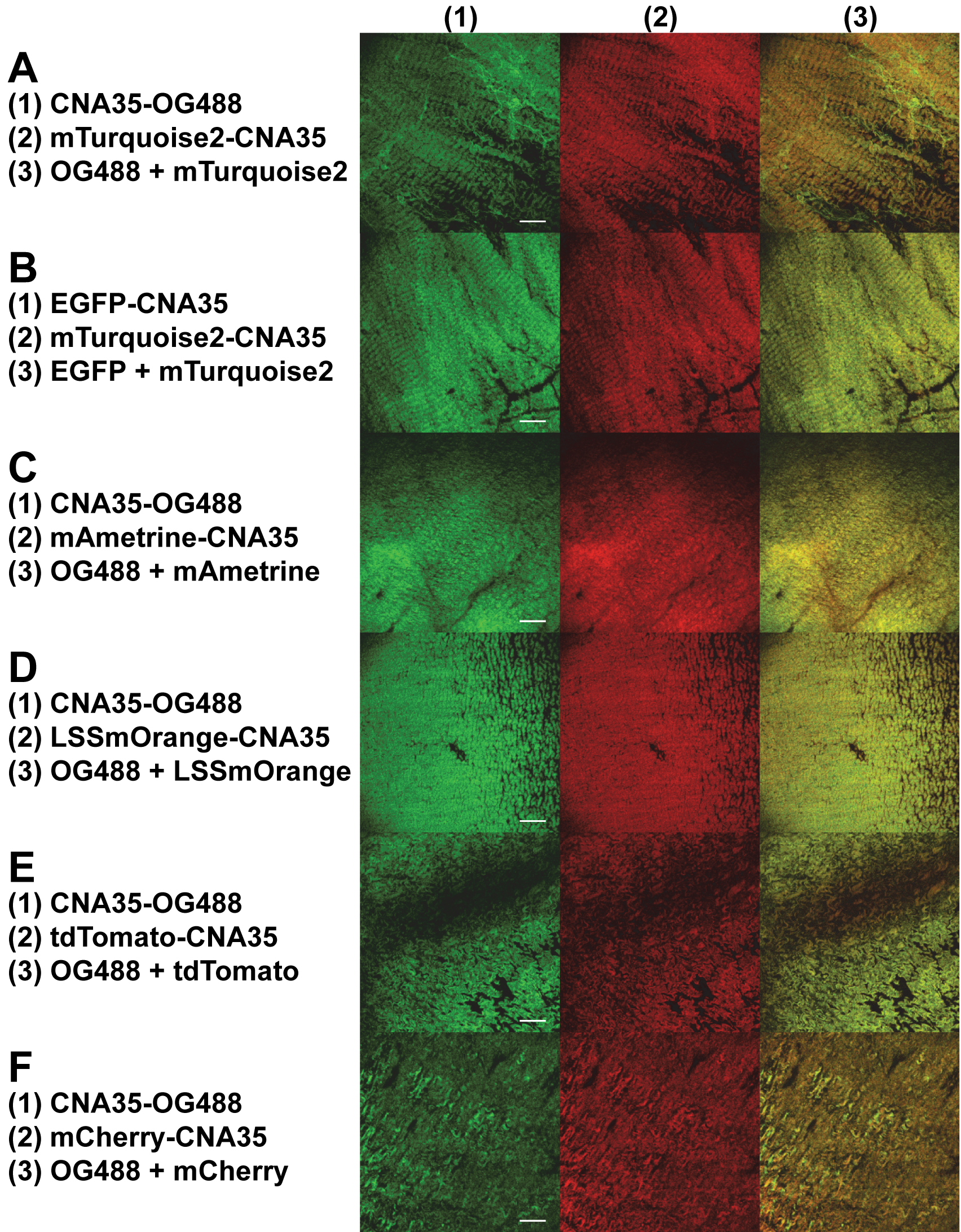

Supplement: S14 Figure — Dual staining of porcine pericardial tissue with CNA35-OG488 and FP-CNA35 probes. Confocal fluorescence microscopy images showing dual staining of porcine pericardial tissue with CNA35-OG488 and FP-CNA35 probes. Images of collagen stained with 1 µM CNA35-OG488 and 1 µM mTurquoise2-CNA35 (A), mAmetrine-CNA35 (C), LSSmOrange-CNA35 (D), tdTomato-CNA35 (E) and mCherry-CNA35 (F), after overnight incubation of tissue with the probes in PBS at room temperature. Left: OG488 emission. Middle: FP emission. Right: OG488+ FP emission. (B) EGFP-CNA35 was co-stained with mTurquoise2-CNA35, as spectra of OG488 and CNA35 show too much overlap. Excitation wavelengths and spectral emission windows used for imaging can be found in table S3. Scale bar = 100 µm. (TIF) [file pone.0114983.s014.tif]

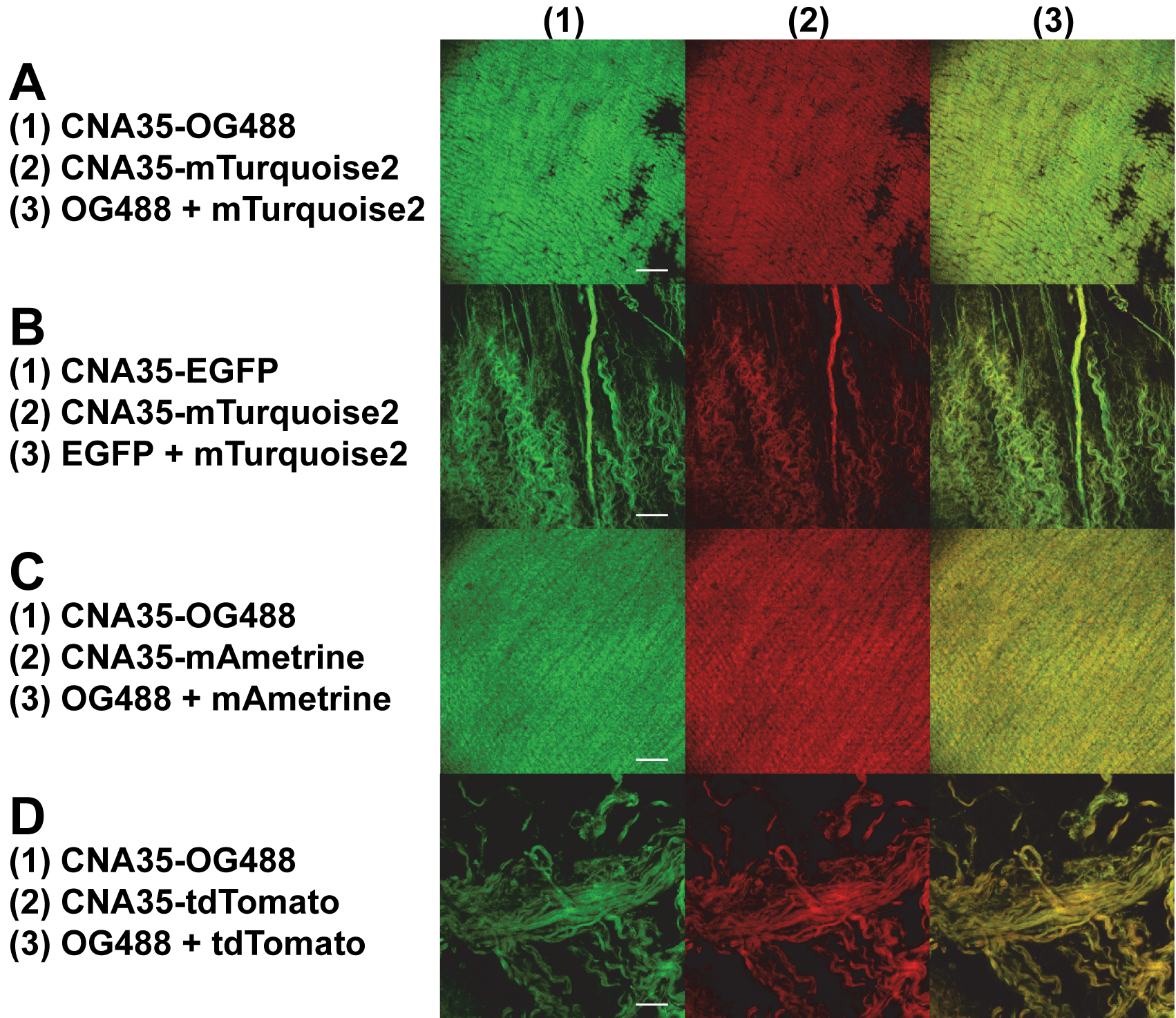

Supplement: S15 Figure — Dual staining of porcine pericardial tissue with CNA35-OG488 and CNA35-FP probes. Confocal fluorescence microscopy images showing dual staining of porcine pericardial tissue with CNA35-OG488 and CNA35-FP probes. Images of collagen stained with 1 µM CNA35-OG488 and 1 µM CNA35-mTurquoise2 (A), CNA35-mAmetrine (C) and CNA35-tdTomato (D), after overnight incubation of tissue with the probes in PBS at room temperature. Left: OG488 emission. Middle: FP emission. Right: OG488+ FP emission. (B) CNA35-EGFP was co-stained with CNA35-mTurquoise2, as spectra of OG488 and EGFP show too much overlap. Excitation wavelengths and spectral emission windows used for imaging can be found in table S3. Scale bar = 100 µm. (TIF) [file pone.0114983.s015.tif]
