# Supplementary material for: Colorful Protein-Based Fluorescent Probes for Collagen Imaging
Source: PLoS One. 2014 Dec 9;9(12):e114983. doi: 10.1371/journal.pone.0114983 (PMC4260915; doi:10.1371/journal.pone.0114983)
Supplement: S1 Table — Primers used for cloning of CNA35 gene into pET28a vector. Restriction sites for NheI and EcoRI in primer CNA35_to_pET28a FW, and for AatII and XhoI in primer CNA35_to_pET28a RV are shown italicized and underlined. (PDF) [file pone.0114983.s016.pdf]

**Table S1. Primers used for cloning of CNA35 gene into pEET28a vector**

| Primer name | Sequence |
|---|---|
| **CNA35_to_ pET28a FW** | 5'-TCATC*GCTAGC*TCAGGTGCA*GAATTC*CACGGATCCGCACGAGATATTTC-3' |
| **CNA35_to_ pET28a RV** | 5'-GATAC*CTCGAG*CAATGCCTTACAG*GACGTC*TGAAGCCTTGGTATCTTTATCCTG-3' |
