# Supplementary material for: Colorful Protein-Based Fluorescent Probes for Collagen Imaging
Source: PLoS One. 2014 Dec 9;9(12):e114983. doi: 10.1371/journal.pone.0114983 (PMC4260915; doi:10.1371/journal.pone.0114983)
Supplement: S2 Table — Primers used for cloning fluorescent protein gene at N- or C-terminus of CNA35 gene. In the primers used for constructing N-terminal fusions of fluorescent protein to CNA35, restriction sites for NheI and EcoRI are shown italicized and underlined in forward and reverse primer, respectively. In the primers used for constructing C-terminal fusions of fluorescent protein to CNA35, restriction sites for AatII and XhoI are shown italicized and underlined in forward and reverse primer, respectively. (PDF) [file pone.0114983.s017.pdf]

Table S2. Primers used for cloning fluorescent protein gene at N- or C-terminus of CNA35 gene

Primer name	Sequence
**mTurquoise2-CNA35 FW**	5'-ATAAT*GCTAGC*CCGGTCGCCACCATG-3'
**mTurquoise2-CNA35 RV**	5'-TAGTA*GAATTC*CTTGTACAGCTCGTCCATGCC-3'
**EGFP-CNA35 FW**	5'-ATATA*GCTAGC*ATGGTGAGCAAGGGCGAG-3'
**EGFP-CNA35 RV**	5'-ATGAT*GAATTC*TCCGGACTTGTACAGCTCG-3'
**mAmetrine-CNA35 FW**	5'-TTAAT*GCTAGC*CCGGTCGCCACCATGG-3'
**mAmetrine-CNA35 RV**	5'-AATAT*GAATTC*GAGAGTGATCCCGGCGG-3'
**LSSmOrange-CNA35 FW**	5'-TTAAA*GCTAGC*ATGGTGAGCAAAGGTGAGG-3'
**LSSmOrange-CNA35 RV**	5'-TATAT*GAATTC*TTTATACAGTTCGTCCATACCGC-3'
**tdTomato-CNA35 FW**	5'-ATATA*GCTAGC*GAGGTCGATGGTATGGTGAGC-3'
**tdTomato-CNA35 RV**	5'-TACGT*GAATTC*CTTGTACAGCTCGTCCATGCC-3'
**mCherry-CNA35 FW**	5'-ATATA*GCTAGC*ATGGTTTCTAAGGGCGAAGAGG-3'
**mCherry-CNA35 RV**	5'-TATAT*GAATTC*CTTGTATAACTCATCCATGCCACCG-3'
**CNA35-mTurquoise2 FW**	5'-ATAAT*GACGTC*ACCATGGTGAGCAAGGGCG-3'
**CNA35-mTurquoise2 RV**	5'-TAGTA*CTCGAG*ATCTTACTTGTACAGCTCGTCCATGCC-3'
**CNA35-EGFP FW**	5'-ATATA*GACGTC*ATGGTGAGCAAGGGCGAG-3'
**CNA35-EGFP RV**	5'-ATGAT*CTCGAG*TTATCCGGACTTGTACAGCTCG-3'
**CNA35-mAmetrine FW**	5'-TTAAT*GACGTC*CCGGTCGCCACCATGG-3'
**CNA35-mAmetrine RV**	5'-AATAT*CTCGAG*TTAGAGAGTGATCCCGGCGG-3'
**CNA35-LSSmOrange FW**	5'-TATTA*GACGTC*ATGGTGAGCAAAGGTGAGG-3'
**CNA35-LSSmOrange RV**	5'-TATAT*CTCGAG*TTATTTATACAGTTCGTCCATACCGC-3'
**CNA35-tdTomato FW**	5'-ATATA*GACGTC*GGCAGTGGCAGCGGAGATG-3'
**CNA35-tdTomato RV**	5'-TATAT*CTCGAG*ATCGTTACTTGTACAGCTCGTCCATGCC-3'
**CNA35-mCherry FW**	5'-ATATA*GACGTC*ATGGTTTCTAAGGGCGAAGAGG-3'
**CNA35-mCherry RV**	5'-TAGAT*CTCGAG*ATATTACTTGTATAACTCATCCATGCCACCG-3'
