# Supplementary material for: Colorful Protein-Based Fluorescent Probes for Collagen Imaging
Source: PLoS One. 2014 Dec 9;9(12):e114983. doi: 10.1371/journal.pone.0114983 (PMC4260915; doi:10.1371/journal.pone.0114983)
Supplement: S3 Table — Excitation and emission wavelengths used for imaging pericardial tissue stained with probe #1 and #2. (PDF) [file pone.0114983.s018.pdf]

Table S3. Excitation and emission wavelengths used for imaging pericardial tissue stained with probe #1 and #2

CNA35-FP[a] #1	Excitation wavelength (nm)	Emission wavelength (nm)	CNA35-FP[a] #2	Excitation wavelength (nm)	Emission wavelength (nm)
**OG488**	990[b]	499-539	**mTurquoise2**	870[b]	454-494
**EGFP**	976[b]	487-527	**mTurquoise2**	870[b]	454-494
**OG488**	495[c]	499-539	**mAmetrine**	812[b]	506-546
**OG488**	990[b]	499-539	**LSSmOrange**	874[b]	552-592
**OG488**	495[c]	499-539	**tdTomato**	554[c]	590-630
**OG488**	990[b]	499-539	**mCherry**	587[c]	590-630

[a] Or FP-CNA35.

[b] Excitation using two-photon laser.

[c] Excitation using white light laser.
